# CERS6 promotes esophageal squamous cell carcinoma proliferation by increasing the stability of RPN1

**DOI:** 10.1038/s41420-025-02727-y

**Published:** 2025-11-07

**Authors:** Wenjing Chen, Yuxin Zhai, Xiaoxiao Yang, Weizhe Zhang, Dan Gao, Xiaokun Zhao, Nannan Zhao, Yuhan Zhang, Qiang Yuan, Zhiying Zhao, Qiong Wu, Yifei Xie, Jimin Zhao, Zigang Dong, Kangdong Liu, Yanan Jiang

**Affiliations:** 1 State Key Laboratory of Metabolic Dysregulation & the Prevention and Treatment of Esophageal Cancer, School of Basic Medical Sciences, Zhengzhou University, Zhengzhou, Henan China; 2 China-US (Henan) Hormel Cancer Institute, Zhengzhou, Henan China; 3 Department of Pathophysiology, School of Basic Medical Sciences, Zhengzhou University, Zhengzhou, Henan China; 4 Tianjian Laboratory of Advanced Biomedical Sciences, Zhengzhou, Henan China; 5 Department of Neurosurgery, The First Affiliated Hospital of Zhengzhou University, Zhengzhou, Henan China; 6 Henan International Joint Laboratory of Cancer Chemoprevention, Zhengzhou University, Zhengzhou, Henan China

**Keywords:** Oesophageal cancer, Cancer

## Abstract

Esophageal squamous cell carcinoma (ESCC) is associated with poor prognosis because it is typically diagnosed at a moderate or advanced stage. Investigating the precise molecular mechanism of ESCC pathogenesis is essential for developing new therapeutic strategies. In this study, we demonstrated that ceramide synthase 6 (CERS6) was overexpressed and correlated with a worse prognosis in ESCC. Moreover, CERS6 promoted ESCC cell proliferation in vitro and in vivo. Mechanistically, CERS6 sustained the stability of ribophorin 1 (RPN1) by inhibiting its ubiquitination. Subsequently, CERS6 reduced endoplasmic reticulum (ER) stress and reactive oxygen species (ROS) by activating the RPN1–inositol-requiring enzyme 1 (IRE1)–X-box binding protein 1 (XBP1) signaling pathway. Interestingly, the antisense oligonucleotides (ASOs) targeting CERS6 inhibited the growth of ESCC through the RPN1-IRE1-XBP1 signaling pathway. Collectively, our study reveals an unprecedented function and mechanism of CERS6, which is distinct from ceramide synthases, in the development of ESCC, highlighting its potential as a promising therapeutic target.

## Introduction

Esophageal cancer (EC) is the seventh most commonly occurring cancer, ranking sixth in mortality rates [1, 2]. It is classified into two primary types: esophageal squamous cell carcinoma (ESCC) and esophageal adenocarcinoma (EAC) [3, 4]. Surgery, radiotherapy, and chemotherapy are the conventional treatments for ESCC patients. Recently, molecular targeted therapy, mainly targeting EGFR, HER2, and VEGF, and cancer immunotherapy, especially the combination of PD-L1-based immunotherapy with other targeted therapies, are promising approaches [5–7]. However, despite these advancements, the prognosis for ESCC patients remains poor due to the limited effectiveness of these therapies [5, 8, 9]. Therefore, it is crucial to understand the molecular mechanism of ESCC pathogenesis to develop more effective treatments.

Accumulating evidence shows that endoplasmic reticulum (ER) stress is significantly related to ESCC pathogenesis [10–15]. For instance, alpha-inducible protein 6 (IFI6) depletion inhibits ESCC progression through reactive oxygen species (ROS) accumulation via mitochondrial dysfunction and ER stress. Additionally, ACP-5862 suppresses the growth of ESCC by activating ER stress and generating ROS, ultimately inducing apoptosis [16]. Accumulating evidence indicates that ROS plays a vital role in regulating aggressive cancers, and targeting ER stress may be a potential therapeutic strategy [17–19].

Ceramide synthase 6 (CERS6), located in the endoplasmic reticulum, is one of the ceramide synthases, which generate ceramides with distinct acyl chain lengths. It primarily generates C16-ceramide as part of the sphingolipid metabolic pathway [20]. Recent research has shown that CERS6 plays a significant role in the development of cancer. In pancreatic cancer, CERS6 contributes to the accumulation of mutat p53 due to the production of C16-ceramide [21]. CERS6 has also been identified as an oncoprotein in gastric cancer that disrupts the SOCS2/JAK2/STAT3 pathway [22]. Several studies have indicated that CERS6 promotes lamellipodia formation of cells, which is essential for lung cancer migration and metastasis [23, 24]. Furthermore, CERS6 regulates the calcium pathway and is negatively correlated with disease progression in ovarian cancer [25]. Therefore, targeting CERS6 might be a promising strategy for cancer therapy.

In the current study, we demonstrated that high expression of CERS6 is significantly associated with poor clinical prognosis in patients with ESCC. CERS6 promoted ESCC growth in vitro and in vivo. Remarkably, we discovered that CERS6 interacted with RPN1 through the Tram-Lag-CLN8 (TLC) domain and was essential for sustaining the stability of RPN1. Consequently, CERS6 activated the heat shock protein family A member 5 (HSPA5)–inositol-requiring enzyme 1 (IRE1)–X-box binding protein 1 (XBP1) signaling pathway, thereby modulating ER stress and ROS levels in ESCC cells. Our research has revealed an unprecedented mechanism regarding CERS6’s role, distinct from ceramide synthases, in ESCC development.

## Results

### High CERS6 expression is negatively correlated with ESCC prognosis

**Fig. 1:**
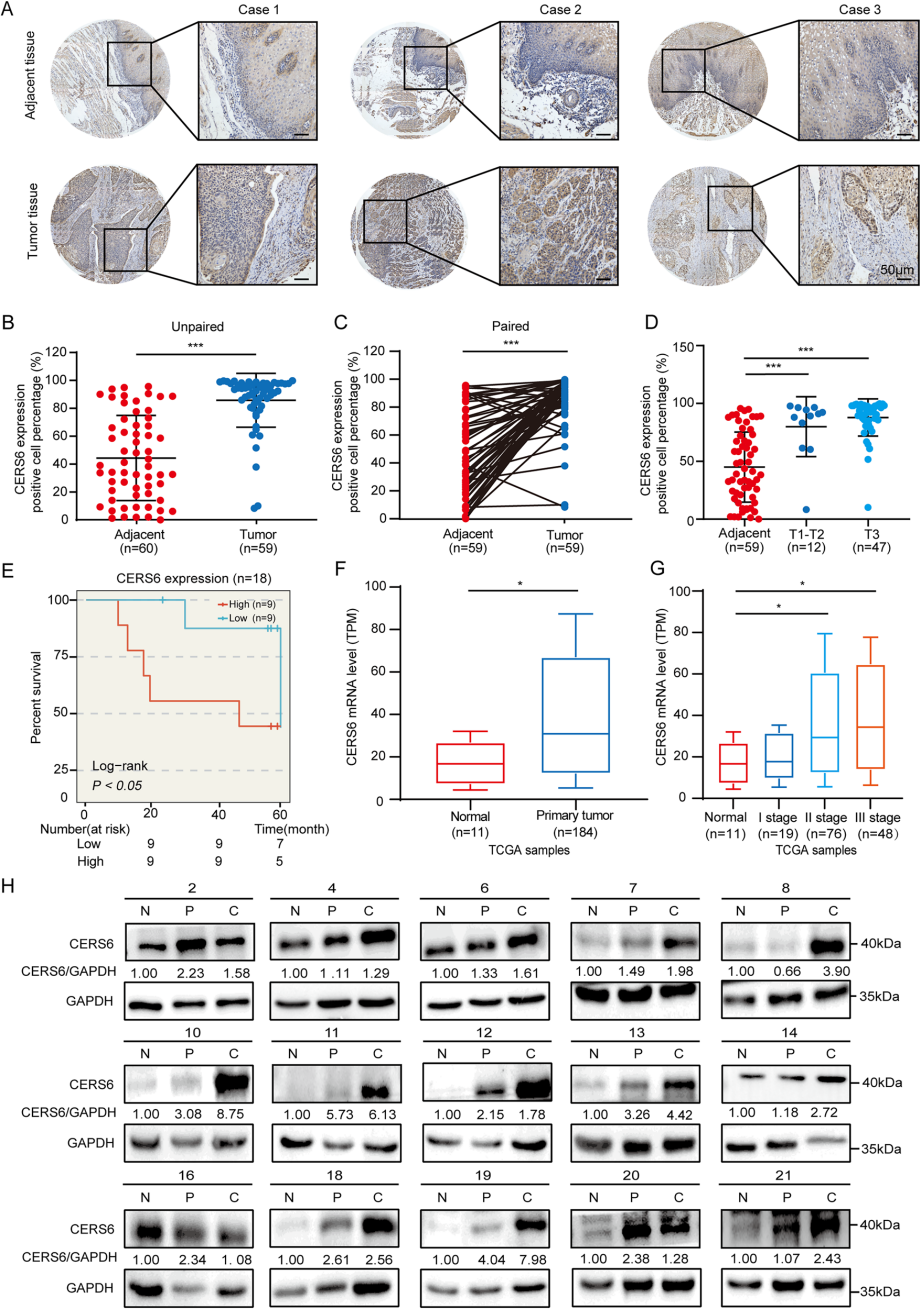
High CERS6 expression is negatively correlated with ESCC prognosis. **A** Representative IHC staining images of ESCC tissue array using CERS6 antibody in adjacent tissues and tumor tissues. Scale bar: 50 μm. **B**, **C** Quantification of CERS6 expression in unpaired (**B**) and paired (**C**) tissues. **D** Protein levels of CERS6 in adjacent, T1-T2 and T3 ESCC tissues. **E** Kaplan-Meier analysis of the relationship between CERS6 expression and the prognosis of ESCC patients from the tissue microarray. **F** CERS6 mRNA levels in esophageal cancer and normal esophageal tissue from the TCGA database. **G** The mRNA level of CERS6 was analyzed based on tumor histology using the data from the TCGA database. **H** The expression of CERS6 in 15 paired normal (N), peri-tumor (P), and cancerous (C) tissues of ESCC patients by Western blot. *P < 0.05, **P < 0.01, ***P < 0.001.

To explore the clinicopathological significance of CERS6 in ESCC, we examined its protein levels of CERS6 in a tissue microarray by immunohistochemistry (IHC). The expression of CERS6 in ESCC was primarily in the cytoplasm and nucleus with various brown-yellow intensities. The level of immunostaining positivity in ESCC tissues was notably higher than that of the adjacent tissues (Fig. 1A). Furthermore, quantitative analysis showed that the average positive percentage of CERS6 in ESCC and adjacent tissues was 85.58% and 44.39%, respectively (Fig. 1B). The paired analysis showed that CERS6 protein levels were higher in ESCC than in adjacent tissues (Fig. 1C). These results indicated that CERS6 is overexpressed in ESCC tissues. Subsequently, we analyzed the relationship between CERS6 protein levels and clinical information of patients with ESCC. We found that CERS6 protein levels were significantly increased in T1-T2 and T3 ESCC compared to the adjacent tissues (Fig. 1D) (45.02% in adjacent tissues, 79.91% in T1-T2, and 87.52% in T3). However, no significant difference in CERS6 expression was observed among different T stages of ESCC (Fig. S1A). Subsequently, ESCC patients were stratified into low and high CERS6 expression groups based on the median positive percentage. We investigated whether CERS6 expression was associated with the prognosis of ESCC by the log-rank test and found that patients with high CERS6 expression had significantly shorter overall survival than those with low CERS6 expression (*P* < 0.05, Fig. 1E) (median survival time was 48 and 61 months, respectively). Next, we examined the relationship between CERS6 protein expression and other characteristics such as gender, age, TNM stage, pathological grade, and lymph node metastasis. The results showed that CERS6 protein expression was not correlated with age (*P* = 0.344), gender (*P* = 0.711), pathological grade (*P* = 0.657), TNM stage (*P* = 0.174), and lymph node metastasis (*P* = 0.683) (Table 1). These results indicate that ESCC patients with high CERS6 have a poorer prognosis.

**Table 1:** Cohort characteristics of CERS6 in ESCC patients.

Clinicopathological characteristics		Number (*n* = 59)	Positive percentage (%)	CERS6		
				Low expression (*n* = 29)	High expression (*n* = 30)	
Age	≤60	17	81.76 ± 23.55	10	7	*P* = 0.344
	>60	42	87.49 ± 17.35	19	23	*χ2* = 0.894
Gender	Male	42	88.47 ± 13.29	20	22	*P* = 0.711
	Female	17	79.34 ± 28.89	9	8	*χ2* = 0.137
Pathology grade	I	6	80.62 ± 24.44	4	2	*P* = 0.657
	II	26	90.57 ± 10.44	12	14	
	III	27	82.12 ± 24.33	13	14	χ2 = 0.841
T classification	T1-T2	12	77.95 ± 28.21	8	4	*P* = 0.174
	T3	47	87.85 ± 16.09	21	26	*χ2* = 1.849
Lymph node	N0	33	88.66 ± 13.27	17	16	*P* = 0.683
	N1-N3	26	82.25 ± 24.80	12	14	*χ2* = 0.167

Numbers do not equal the total number due to missing data.

Tumor TNM staging components, including Tumor (T), Node (N), and Metastasis (M), were defined according to the American Joint Committee on Cancer (AJCC) 7th edition.

To further confirm whether the mRNA expression of CERS6 is higher in ESCC, we analyzed the mRNA sequencing data from The Cancer Genome Atlas (TCGA) database, which contains 11 normal esophageal and 184 esophageal cancer tissues. Results showed that the mRNA levels of CERS6 were markedly increased in esophageal cancer tissues compared to normal tissues (Fig. 1F). The mRNA levels of CERS6 were up-regulated in ESCC and EAC (Fig. 1G). Similarly, the mRNA levels of CERS6 were significantly elevated at T1 and T2 compared to normal tissues (Fig. S1B). Interestingly, CERS6 mRNA was up-regulated in 13 other cancer types by analyzing the TCGA and GTEx databases (Fig. S1C). The data indicate that the mRNA expression of CERS6 is higher in ESCC.

Furthermore, the CERS6 protein levels in paired normal (N), peri-tumor (P), and cancerous (C) tissues of 21 ESCC patients were measured by Western blot. We found that the CERS6 protein levels were significantly higher than in peri-tumor and normal tissues (Figs. 1H and S1D). Based on these results, we conclude that CERS6 is higher and is associated with a lower survival percentage in ESCC patients.

### CERS6 promotes cell proliferation of ESCC in vitro and in vivo

**Fig. 2:**
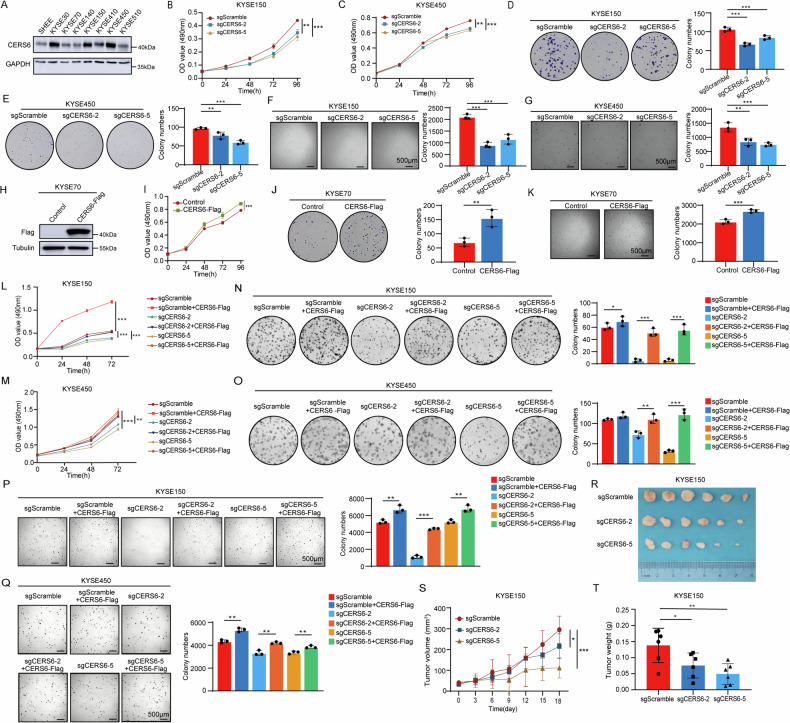
CERS6 promotes cell proliferation of ESCC in vitro and in vivo. **A** CERS6 protein level in SHEE and ESCC cells. **B**, **C** Cell viability of KYSE150 (**B**) and KYSE450 (**C**) cells with stable CERS6 knockout was measured by MTT assay. **D**, **E** The representative plate clone images of stably knocking out CERS6 in KYSE150 cells (**D**) and KYSE450 cells (**E**) were obtained by a plate clone formation assay and the experimental results were statistically analyzed. **F**, **G** In the soft agar experiment, the representative clone images of stably knocking out CERS6 in KYSE150 (**F**) and KYSE450 (**G**) cells were obtained, and the number of clones was statistically analyzed. Scale bar: 500 μm. **H** The overexpression efficiency of CERS6 in KYSE70 cells was detected by Western blot. **I** The proliferative ability of KYSE70 cells after CERS6 overexpression was detected by the MTT assay. **J** Representative plate clone images and statistical analysis of KYSE70 after CERS6 overexpression. **K** Representative clone images and statistical analysis of overexpressed CERS6 in KYSE70 cells in soft agar assay. Scale bar: 500 μm. **L**, **M** The soft agar assay results and statistical analysis of cell viability after rescuing CERS6 in sgCERS6 KYSE150 (**L**) and KYSE450 (**M**) cells by MTT assay. **N**, **O** The representative plate clone images of rescued CERS6 in KYSE150 (**N**) and KYSE450 (**O**) sgCERS6 cells were obtained by a plate clone formation assay, and the cell proliferation ability after rescued CERS6 was verified. **P**, **Q** In the soft agar experiment, the representative clone images and statistical analysis of CERS6 were restored in KYSE150 (**P**) and KYSE450 (**Q**) sgCERS6 cells (Scale bar: 500 μm). The experimental results were statistically analyzed. **R** Tumor image of CDX model in sgCERS6 KYSE150 cell group (*n* = 6). **S** Tumor volumes were measured and calculated at specified intervals in the sgCERS6 KYSE150 cell group. **T** The weights of tumor xenografts in the sgCERS6 KYSE150 cell were measured. *P < 0.05, **P < 0.01, ***P < 0.001.

To analyze the role of CERS6 in ESCC, the protein levels of CERS6 were measured in seven ESCC cell lines (KYSE30, KYSE70, KYSE140, KYSE150, KYSE410, KYSE450, KYSE510) and an immortalized esophageal cell line (SHEE) using Western blot. The results revealed that CERS6 protein levels were higher in KYSE30, KYSE150, KYSE410, and KYSE450 cells than in SHEE cells. However, KYSE70 cells had a lower CERS6 protein level than other ESCC cells (Fig. 2A). Next, CERS6 was knocked out and verified in KYSE150 (Fig. S2A) and KYSE450 cells (Fig. S2B). The proliferative ability of the cells was then evaluated using MTT assays. In KYSE150 cells, the proliferation of sgCERS6-2 and sgCERS6-5 cells was reduced by 20.5% and 29.5% at 96 h, respectively (Fig. 2B). In KYSE450 cells, the proliferation of sgCERS6-2 and sgCERS6-5 cells decreased by 14% and 17% at 96 h, respectively (Fig. 2C). The results showed that the proliferation capacity of KYSE150 and KYSE450 cells decreased after CERS6 was knocked out. We then evaluated the anchor-dependent growth ability by a plate clone formation assay. The number of colonies was reduced by 37.5% and 20.5% in KYSE150, and by 42.1% and 72.8% in KYSE450 cells for sgCERS6-2 and sgCERS6-5, respectively (Fig. 2D, E). We subsequently evaluated the anchor-independent growth ability using a soft agar assay. In KYSE150 cells, the number of clones in the sgCERS6-2 and sgCERS6-5 groups decreased 57.3% and 45.3%, respectively (Fig. 2F); in KYSE450 cells, the number of clones decreased 38.1% and 45.2%, respectively (Fig. 2G). Therefore, we conclude that the knockout of CERS6 significantly suppresses the proliferation and clonogenicity of ESCC cells.

Subsequently, we investigated the effect of CERS6 overexpression on the proliferation of KYSE70 cells with low CERS6 expression. A pLVX-IRES-Puro-3×Flag plasmid containing the full-length CERS6 was transfected into KYSE70 cells (Fig. 2H). By MTT assay, we found that the OD values of KYSE70 cells increased by 14.4% at 96 h after CERS6 overexpression (Fig. 2I), *P* < 0.05. Additionally, the number of clones in CERS6-overexpressed KYSE70 cells was increased to 121.4% by a plate clone formation assay (Fig. 2J), *P* < 0.05. The results of the soft agar assay showed that the clone number of CERS6-overexpressed KYSE70 cells had increased by 26.7% (Fig. 2K), *P* < 0.05. These results demonstrate that high expression of CERS6 enhances cell proliferation in ESCC.

Based on these results, we designed a rescue assay to determine whether restoring CERS6 expression in KYSE150 and KYSE450 CERS6 knockout cells would regain its function. Western blot results showed that the expression of CERS6 protein in KYSE150 (Fig. S2C) and KYSE450 (Fig. S2D) cells increased in the rescue assay. The MTT assay results showed that after restoring CERS6 in sgCERS6 KYSE150 (Fig. 2L) and KYSE450 (Fig. 2M) cells, the proliferation ability of the CERS6 rescuing cells was significantly improved at 72 h, while the proliferation ability of the CERS6 knockout cells was significantly inhibited. Next, we tested the anchor-dependent growth ability using a plate clone formation assay. The number of clones increased when rescuing CERS6 in the knocked-out CERS6 KYSE150 (Fig. 2N) and KYSE450 (Fig. 2O) cells. Moreover, the colony numbers increased dramatically in rescuing CERS6 KYSE150 (Fig. 2P) and KYSE450 (Fig. 2Q) cells by a soft agar assay. Collectively, our results demonstrate that high CERS6 facilitates ESCC progression in vitro.

To explore the function of CERS6 in vivo, KYSE150 cells with stable CERS6 knockout were used for cell-derived xenograft (CDX) models. The tumor xenografts were significantly smaller in sgCERS6 KYSE150 cells (Fig. 2R). We also found that the tumor volume of KYSE150 (Fig. 2S) xenografts significantly decreased after CERS6 was knocked out. Moreover, the final weights of CERS6 knockout KYSE150 xenografts were significantly lower than those of control groups (Fig. 2T). These findings confirm that CERS6 promotes tumor growth in the ESCC CDX models.

### CERS6 binds with RPN1 in ESCC cells

**Fig. 3:**
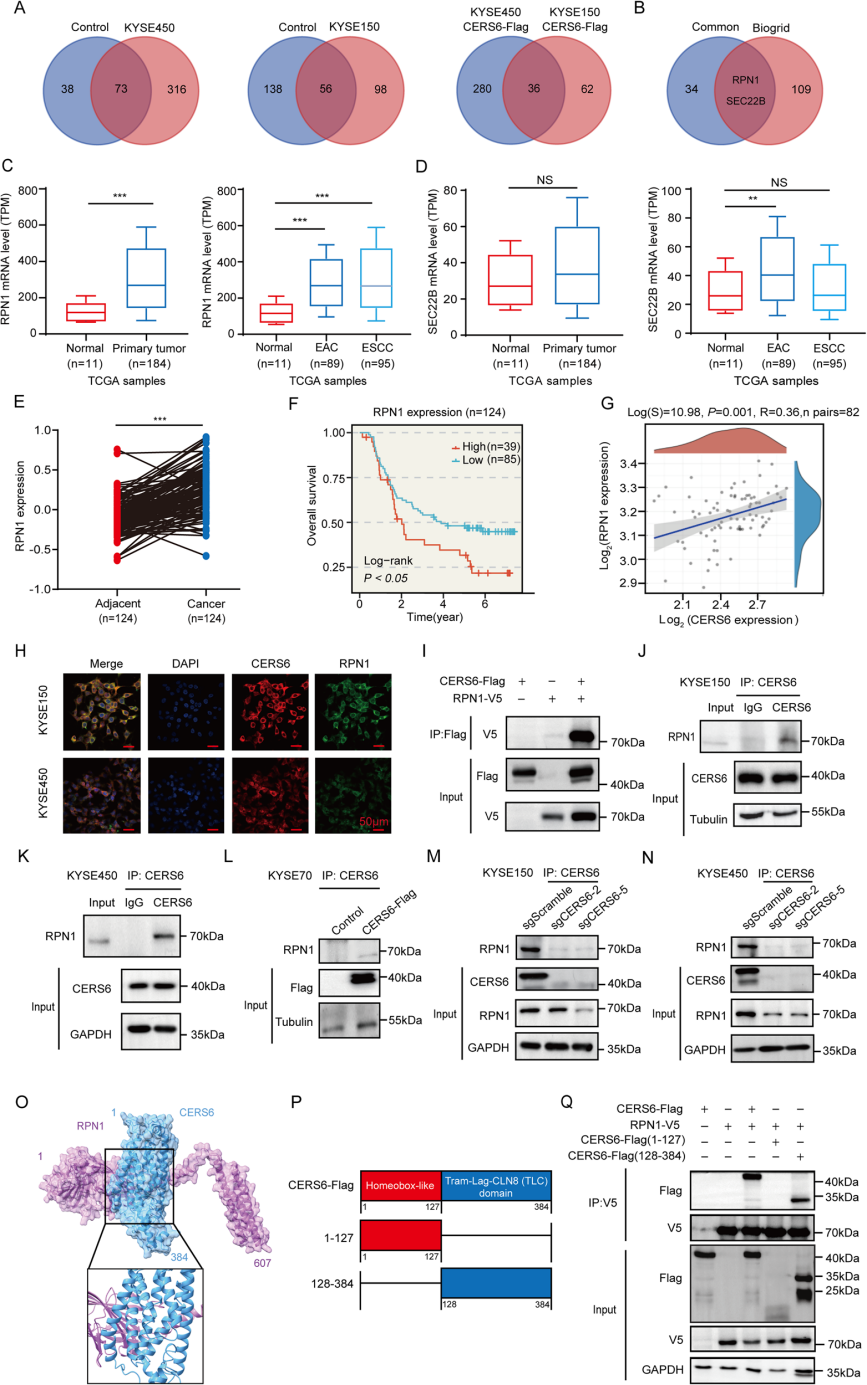
CERS6 binds with RPN1 in ESCC cells. **A** Mass spectrometry results and analysis of the proteins binding with CERS6 in KYSE150 and KYSE450 cells. **B** Mass spectrometry results were combined with the BioGRID database to identify the binding proteins. **C** The RPN1 mRNA levels from TCGA based on esophageal cancer and normal esophageal tissue. The mRNA level of RPN1 in esophageal carcinoma was analyzed based on tumor histology from the TCGA database. **D** The SEC22B mRNA levels from TCGA based on esophageal cancer and normal esophageal tissue. The mRNA level of SEC22B in esophageal carcinoma was analyzed based on tumor histology from the TCGA database. **E** RPN1 protein levels in EC tumors and adjacent tissues were assessed using proteomic data from paired samples from 124 patients with clinical information. **F** Kaplan–Meier analysis of RPN1 in proteomic data from the 124 EC patients with clinical information. **G** A correlation diagram between CERS6 and RPN1 from the Clinical Health Trust Home database (https://www.aclbi.com/). **H** Co-localization of CERS6 and RPN1 in KYSE150 and KYSE450 cells. Photographs were taken under a fluorescence microscope, Scale bar: 50 μm. **I** CERS6 interacted with RPN1 in HEK293 T cells. **J**, **K** CERS6 interacted with RPN1 by the Co-IP assays in KYSE150 (**J**) and KYSE450 (**K**) cells. **L** CERS6 interacted with RPN1 by the Co-IP assays in overexpressed CERS6 KYSE70 cells. **M**, **N** CERS6 interacted with RPN1 by the Co-IP assays after CERS6 knockout in KYSE150 (**M**) and KYSE450 (**N**) cells. **O** The predicted binding model between CERS6 and RPN1 using a computational docking model. **P** Design of truncated CERS6. **Q** Co-Immunoprecipitation assay to detect the combination of truncated CERS6 and RPN1.

To explore the molecular mechanism of CERS6 in ESCC, the proteins interacting with CERS6 were analyzed by pull-down assay combined with mass spectrometry analysis in KYSE150 and KYSE450 cells (Fig. S3A). 316 proteins were obtained in KYSE450 cells, and 98 potential proteins were obtained in KYSE150 cells. We crossed them using Venn analysis (http://bioinformatics.psb.ugent.be/webtools/Venn/), and 36 proteins were enriched (Fig. 3A). Next, we retrieved 111 CERS6-related proteins from the BioGRID database (https://thebiogrid.org/). We cross-referenced the 36 proteins with the 111 proteins in the BioGRID database using Venn analysis, and 2 proteins were identified for further research: Ribophorin I (RPN1) and SEC22 homolog B, Vesicle-trafficking protein SEC22b (SEC22B) (Fig. 3B). RPN1 is an endoplasmic reticulum translocon-associated subunit in the oligosaccharyltransferase (OST) complex and significantly enhances the N-glycosylation of selected membrane proteins [26, 27]. Knockdown of RPN1 has been shown to reduce the proliferation and invasion of HCC cells [28] and breast cancer cells [29]. SEC22B is a component of soluble N-ethylmalimide-sensitive factor attachment protein receptors (SNAREs) that mediate membrane fusion processes in many physiological and pathological pathways [30–33]. We explored the expression of RPN1 and SEC22B in esophageal cancer. According to the TCGA database, RPN1 exhibited significantly higher mRNA levels in esophageal cancer, including EAC and ESCC, compared to normal esophageal tissues (Fig. 3C). In contrast, SEC22B expression showed no significant difference (Fig. 3D).

Subsequently, proteomic data from 124 EC patients were used to analyze the relationship between RPN1 and SEC22B protein expression and the prognosis of esophageal cancer [34]. We found that the protein levels of RPN1 in EC tissues were higher than in normal tissues (Fig. S3B). Moreover, there were significant differences between EC and normal tissues when compared in pairs (Fig. 3E). Furthermore, high expression of RPN1 was positively correlated with poor prognosis in patients with EC (Fig. 3F). In contrast, SEC22B was not associated with the prognosis of EC (*P* > 0.05) (Fig. S3C). The above results indicated that RPN1, not SEC22B, played a vital role in the proliferation of EC cells. Next, the correlations between CERS6 and RPN1 or SEC22B were evaluated in the Clinical Health Trust Home database (https://www.aclbi.com/), and there was a positive correlation between CERS6 and RPN1 (Fig. 3G) or SEC22B (Fig. S3D), with an R-value of 0.36 or 0.29, respectively. Therefore, we hypothesize that RPN1 may be the CERS6-bound protein in ESCC.

To evaluate whether CERS6 and RPN1 were co-localized in ESCC cells, a cell immunofluorescence assay was performed. CERS6 and RPN1 were both localized in the cytoplasm with red and green fluorescence, respectively, in KYSE150 and KYSE450 cells (Fig. 3H), suggesting that CERS6 and RPN1 were co-localized in the cells. To further validate whether RPN1 was a potential binding partner of CERS6, we transfected pLVX-IRES-Puro-3×Flag CERS6 and pcDNA3.1-V5 RPN1 plasmids into HEK293T cells. The results suggested exogenously expressed CERS6 bound to RPN1 in cells (Fig. 3I). Next, we performed Co-immunoprecipitation (Co-IP) assays in ESCC cells. The results showed that CERS6 could bind to RPN1 in KYSE150 (Fig. 3J) and KYSE450 (Fig. 3K) cells. Furthermore, the binding of RPN1 protein was augmented in CERS6-overexpressed cells (Fig. 3L), while it was reduced in KYSE150 (Fig. 3M) and KYSE450 (Fig. 3N) cells after CERS6 knockout. These results suggest that RPN1 is bound to CERS6 in ESCC cells.

Next, we verified the specific binding region between CERS6 and RPN1. We predicted the binding model between them using a computational docking model. Interestingly, the results showed that CERS6 may bind to RPN1 with a -375.04 docking score (Fig. 3O). Based on the prediction of the computer model, we constructed two truncated plasmids for the Homeobox-like (Hox) domain (1-127) and the TLC domain (128-384) of CERS6 (Fig. 3P). We found that CERS6 lacking the TLC domains didn’t interact with RPN1, while CERS6 with the TLC domains could bind to RPN1 (Fig. 3Q), indicating that RPN1 binds to the TLC domain of CERS6. These findings suggest that CERS6 binds to RPN1 through its TLC domain.

### CERS6 promotes cell proliferation by stabilizing the RPN1 protein in ESCC

**Fig. 4:**
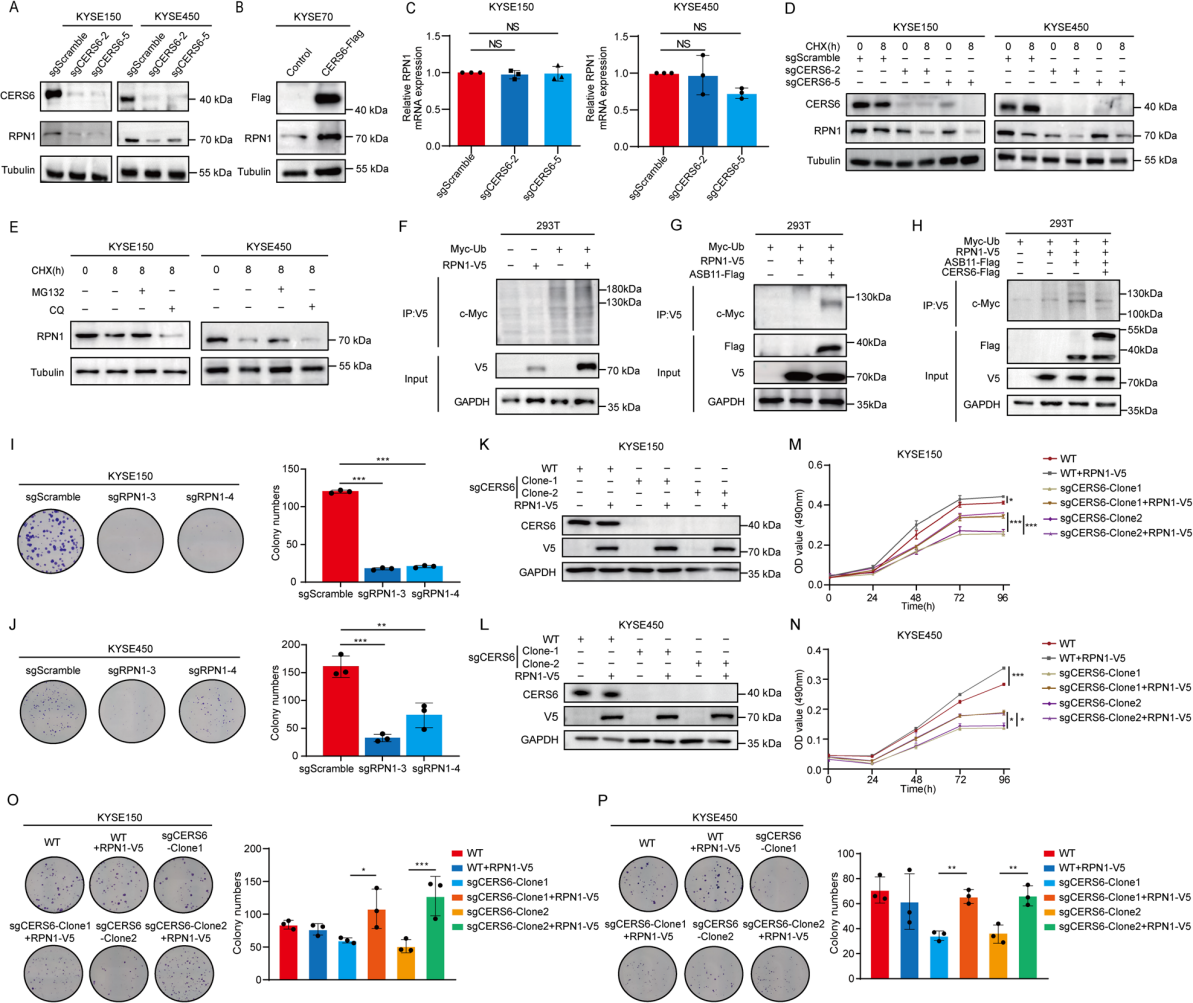
CERS6 promotes cell proliferation by stabilizing the RPN1 protein in ESCC. **A** The level of RPN1 protein in sgCERS6 KYSE150 and KYSE450 cells was detected by Western blot. **B** The protein level of RPN1 in CERS6 overexpressed KYSE70 cells was detected by Western blot. **C** The mRNA level of RPN1 in CERS6 knockout KYSE150 and KYSE450 cells was detected by qPCR. **D** KYSE150 and KYSE450 CERS6-knockout cells were treated with 50 µg/mL CHX. The expression level of RPN1 protein was detected after incubation for 0 h or 8 h. **E** KYSE150 and KYSE450 cells were treated with 50 µg/mL CHX, and incubated with 20 µM CQ and 20 µM MG132 to detect the expression level of RPN1 protein at 0 h and 8 h, respectively. **F** Ubiquitination of RPN1 after transfecting Myc-Ub and RPN1-V5 plasmids in HEK293T cells was determined by ubiquitination assay. **G** Ubiquitination in HEK293T cells after transfecting Myc-Ub, RPN1-V5 and ASB11-Flag plasmids. **H** Ubiquitination of RPN1 after transfecting of Myc-Ub, RPN1-V5, ASB11-Flag and CERS6-Flag plasmids in HEK293T cells. **I**, **J** Representative plate clone images and statistical analysis in KYSE150 (**I**) and KYSE450 (**J**) cells with the stable knockout of RPN1. **K**, **L** Western blot of RPN1 protein levels after rescuing RPN1 in CERS6 knockout KYSE150 (**K**) and KYSE450 (**L**) cells. **M**, **N** Cell viability was detected by MTT assays after rescuing RPN1 in KYSE150 (**M**) and KYSE450 (**N**) sgCERS6 cells. **O**, **P** The representative plate clone images of CERS6 knockout KYSE150 (**O**) and KYSE450 (**P**) cells after rescuing of RPN1 were obtained by a plate clone formation assay, and the proliferation ability of these cells after rescuing of RPN1 was statistically analyzed. *P < 0.05, **P < 0.01, ***P < 0.001.

As the above findings demonstrated that CERS6 was directly bound to RPN1, we further explored how CERS6 regulated RPN1. We found that the protein levels of RPN1 were significantly decreased in KYSE150 and KYSE450 cells after knocking out CERS6 (Fig. 4A). The protein level of RPN1 was elevated in KYSE70 cells overexpressing CERS6 (Fig. 4B). Next, the mRNA levels of RPN1 were measured in KYSE150 and KYSE450 cells after CERS6 knockout. Interestingly, the levels of RPN1 mRNA were not affected (Fig. 4C). These results indicated that CERS6 decreased the stability of the RPN1 protein through post-transcriptional regulation rather than transcriptional regulation. To confirm this, we measured the protein synthesis of CERS6 by cycloheximide (CHX) chase assays at 0 h and 8 h. Compared with 0 h, the protein levels of RPN1 in KYSE150 and KYSE450 cells were significantly decreased after CHX treatment for 8 h (Fig. 4D). These results showed that the CERS6 knockout might induce the degradation of RPN1. To explore how CERS6 regulated the degradation of RPN1 protein, cells were treated with MG132, a proteasome inhibitor, and chloroquine (CQ), a lysosome inhibitor, to ascertain the pathway by which CERS6 knockout regulated the protein degradation of RPN1. The results showed that MG132 (20 µM) significantly inhibited the degradation of RPN1 protein in KYSE150 and KYSE450 cells, while CQ had no effect (Fig. 4E). These results indicate that RPN1 mainly relies on the ubiquitin-proteasome pathway and that elevated levels of CERS6 can prevent the degradation of the RPN1 protein.

Studies have shown that RPN1 is degraded by the ubiquitin-proteasome pathway, and the translocation of RPN1 from the ER to the cytoplasm depends on ubiquitination [35]. We then performed an in vitro ubiquitination assay and found that RPN1 could be ubiquitinated (Fig. 4F). Ankyrin repeat and SOCS box protein 11 (ASB11), an E3 ubiquitin ligase, mediates ubiquitination in the endoplasmic reticulum and interacts with RPN1 to promote its ubiquitination [36]. Moreover, the expression of ASB11 can increase RPN1 protein level in vivo [37], and our results indicated that RPN1 could be ubiquitinated by ASB11 (Fig. 4G). We hypothesized that CERS6 increased the stability of the RPN1 protein by competing with ASB11. To verify this hypothesis, we co-transfected the plasmids CERS6, ASB11, and RPN1 into HEK293T cells and observed that CERS6 inhibited the ubiquitination of RPN1 by ASB11 (Fig. 4H). These results suggest that CERS6 can increase the protein level and stability of RPN1 by restricting the ubiquitin-proteasome pathway.

Next, to evaluate whether the RPN1 protein indeed mediated the oncogenic function of CERS6, we generated RPN1 knockout KYSE150 (Fig. S4A) and KYSE450 (Fig. S4B) cells. Compared with sgScramble, RPN1 protein levels were significantly reduced in both sgRPN1-3 and sgRPN1-4 cells. We investigated the role of RPN1 in ESCC using an MTT assay and a plate clone formation assay in vitro. At 96 h, the proliferation of KYSE150 sgRPN1-3 and sgRPN1-4 cells was inhibited by 59.6% and 59.2%, respectively (Fig. S4C). In KYSE450 cells, the inhibition rates were 89.5% and 54.4%, respectively (Fig. S4D). The results showed that RPN1 depletion inhibits the proliferation of ESCC cells. The results of the plate clone formation assay revealed that the clone numbers of KYSE150 sgRPN1-3, sgRPN1-4 cells were reduced by 85.3%, 82.8% compared to KYSE150 sgScramble cells (Fig. 4I), and the clone numbers of KYSE450 sgRPN1-3, sgRPN1-4 cells decreased by 79.7%, 54.4% compared to sgScramble cells (Fig. 4J). The above results indicate that RPN1 promotes the proliferation of ESCC cells.

We then designed a rescue assay in sgCERS6-KYSE150 (Fig. 4K) and sgCERS6-KYSE450 (Fig. 4L) cells. After RPN1 rescue in sgCERS6-clone1 and sgCERS6-clone2 in KYSE150 (Fig. 4M) and KYSE450 (Fig. 4N) cells, the cell proliferation ability significantly increased by the MTT assay. Furthermore, the cell proliferation ability was also restored in KYSE150 (Fig. 4O) and KYSE450 (Fig. 4P) by plate clone formation assays. The above results suggest that RPN1 promotes ESCC cell proliferation and that the CERS6-RPN1 signaling axis is crucial in facilitating the growth of ESCC cells.

### The CERS6-RPN1 axis inhibits the ROS-mediated apoptosis by the HSPA5-IRE1-XBP1 signaling pathway in ESCC

**Fig. 5:**
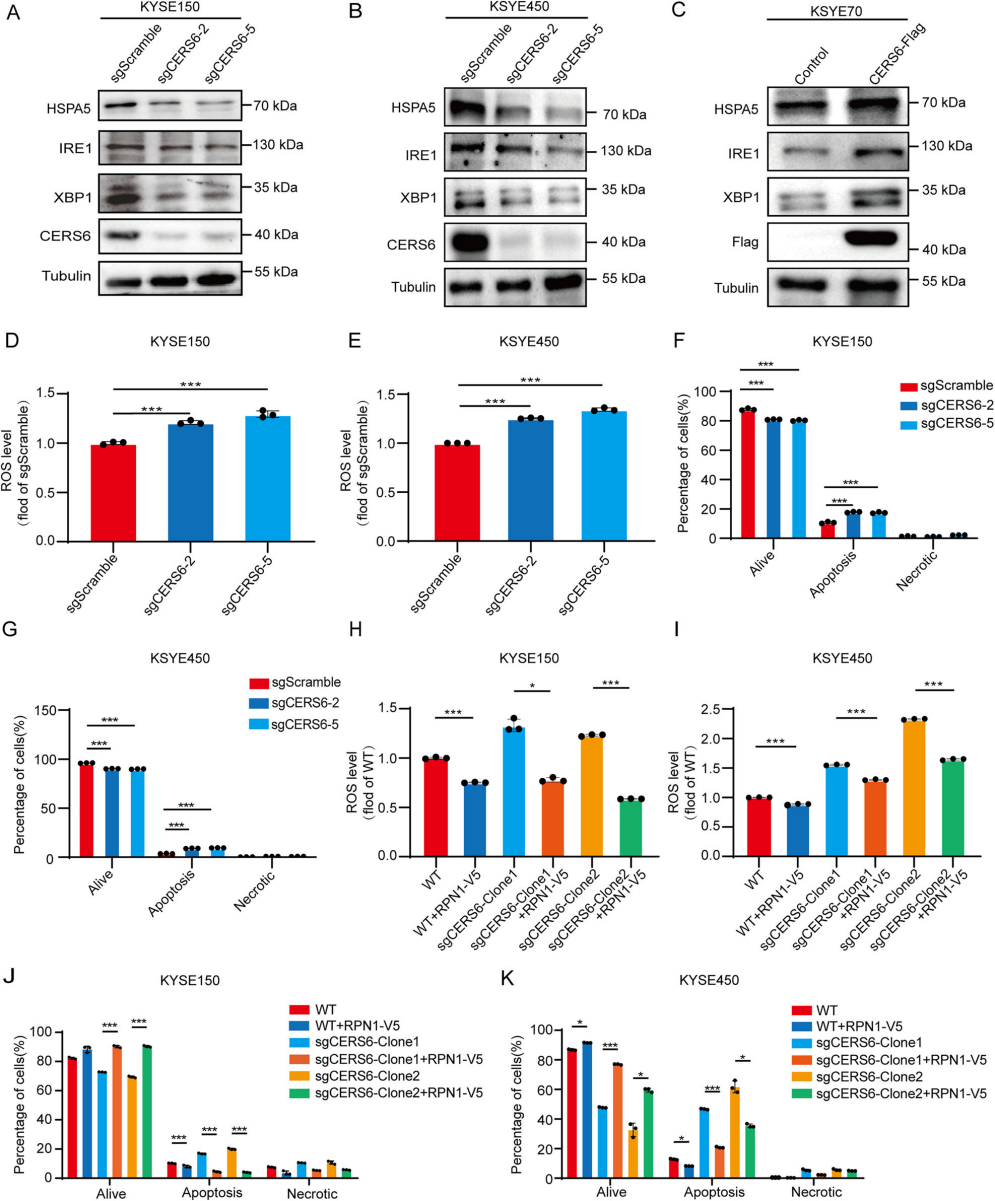
The CERS6-RPN1 axis inhibits the ROS-mediated apoptosis by the HSPA5-IRE1-XBP1 signaling pathway in ESCC. **A**, **B** The ER stress-related protein levels were measured in KYSE150 (**A**) and KYSE450 (**B**) sgScramble, sgCERS6-2, and sgCERS6-5 cells. **C** The ER stress-related protein levels were measured in overexpressed CERS6 KYSE70 cells. **D**, **E** The ROS levels in KYSE150 (**D**) and KYSE 450 (**E**) sgScramble, sgCERS6-2, sgCERS6-5 cells. **F**, **G** The apoptosis assay in KYSE 150 (**F**) and KYSE 450 (**G**) sgScramble, sgCERS6-2, sgCERS6-5 cells. **H**, **I** The ROS levels after rescuing RPN1 in CERS6 knock-out KYSE150 (**H**) and KYSE450 (**I**) cells. **J**, **K** The apoptosis assay after rescuing RPN1 in CERS6 knock-out KYSE150 (**J**) and KYSE450 (**K**) cells. *P < 0.05, **P < 0.01, ***P < 0.001.

Previous studies have shown that CERS6 and RPN1 are related to ER stress. Once CERS6 is knocked down, regulating IRE1, XBP1, and IL-6 in response to myristate is obstructed [38]. The CERS6/C16-ceramide induces apoptosis in human head and neck squamous cell carcinoma by the endoplasmic ER-mediated ATF6/CHOP response pathway [39, 40]. Treatment with primary fibroblast growth factor significantly reduced CERS6/C16-ceramide and ROS levels, and then promoted ER stress-mediated inflammation, oxidative stress, and apoptosis [41]. Moreover, down-regulation of RPN1 inhibited the proliferation and invasion of breast cancer cells and triggered ER stress–induced apoptosis[29]. Therefore, we examined the levels of proteins associated with ER stress after CERS6 knockout. We found that the protein levels of HSPA5, IRE1, and XBP1 decreased in KYSE150 (Fig. 5A) and KYSE450 (Fig. 5B) cells with CERS6 knockout, while they were increased in KYSE70 cells with CERS6 overexpression (Fig. 5C). In addition, ROS levels were higher in sgCERS6-KYSE150 (Figs. 5D and S4A) and sgCERS6-KYSE450 cells (Figs. 5E and S5B) compared to sgScramble cells. Moreover, knocking out CERS6 significantly promoted the apoptosis of KYSE150 (Figs. 5F and S5C) and KYSE450 (Figs. 5G and S5D) cells. Subsequently, we examined the ROS levels after rescuing RPN1 in CERS6 knockout ESCC cells. After rescuing RPN1 in sgCERS6-clone1 and sgCERS6-clone2 in KYSE150 (Figs. 5H and S5E) and KYSE450 (Figs. 5I and S5F) cells, the ROS levels were significantly restored by the ROS assay compared to the CERS6 knockout cells. Moreover, apoptotic cells were also decreased in RPN1-rescued ESCC cells compared with the KYSE150 (Figs. 5J and S5G) and KYSE450 (Figs. 5K and S5H) cells with CERS6 knockout by cell apoptosis assays. Therefore, we conclude that the CERS6-RPN1 axis inhibits ROS-mediated apoptosis through the HSPA5-IRE1-XBP1 signaling pathway in ESCC.

### ASO treatment targeting CERS6 inhibits the cell proliferation of ESCC in vitro and in vivo

**Fig. 6:**
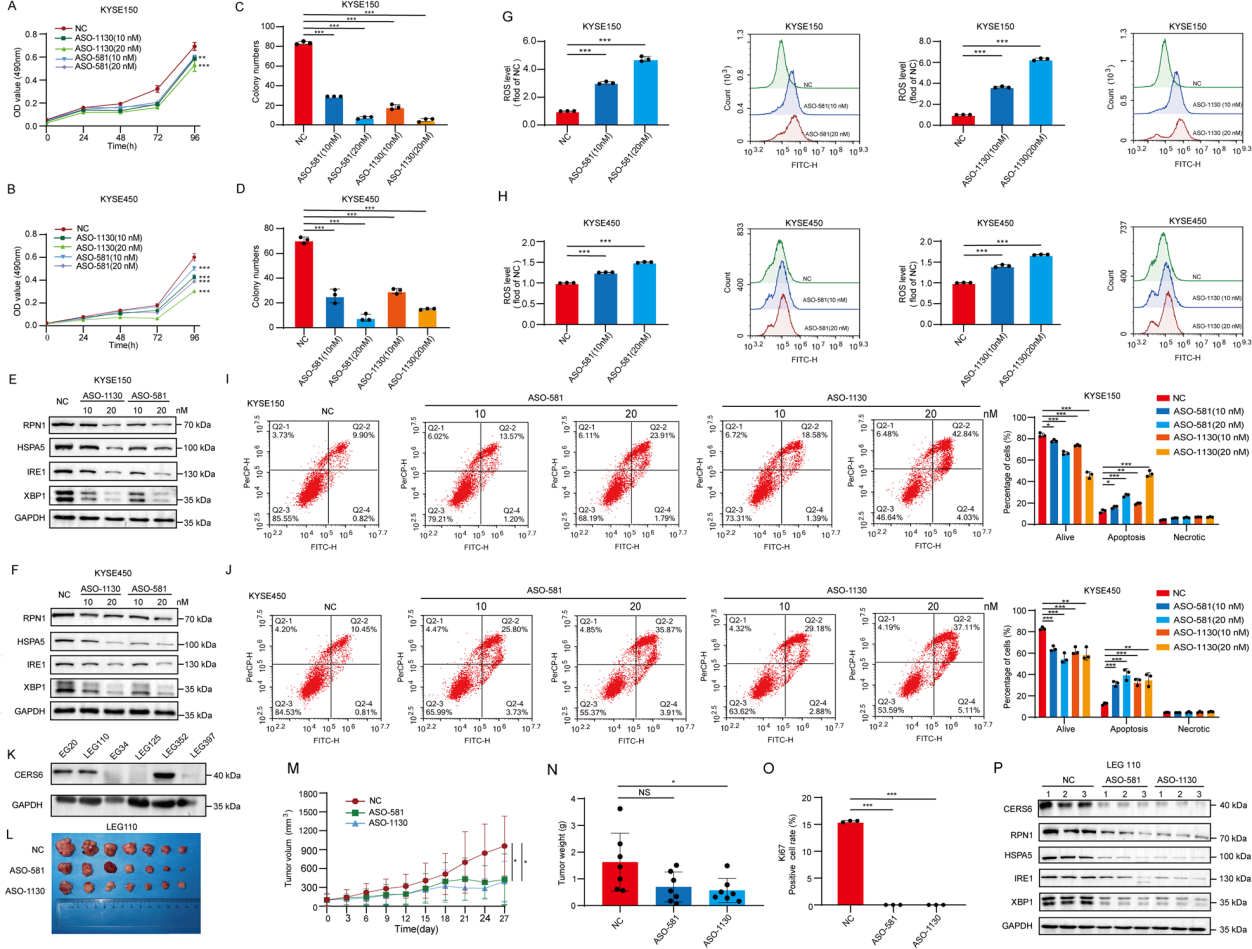
ASO treatment targeting CERS6 inhibits the cell proliferation of ESCC in vitro and in vivo. **A**, **B** Cell viability after ASO treatment targeting CERS6 in KYSE150 (**A**) and KYSE450 (**B**) cells was measured by MTT assay. **C**, **D** The representative plate clone images of cell proliferation ability after ASO treatment for CERS6 in KYSE150 (**C**) and KYSE450 (**D**) cells were obtained by a plate clone formation assay, and the cell proliferation ability after ASO treatment for CERS6 in these cells was statistically analyzed. **E**, **F** The expression of RPN1 protein after ASO treatment targeting CERS6 in KYSE150 (**E**) and KYSE450 (**F**) cells was detected by Western blot. **G**, **H** Representative images of the ROS levels after ASO treatment targeting CERS6 and the ROS levels after ASO treatment targeting CERS6 in KYSE150 (**G**) and KYSE450 (**H**) cells. **I**, **J** The apoptosis assay of ASO treatment targeting CERS6 in KYSE150 (**I**) and KYSE450 (**J**) cells. **K** The protein expression of CERS6 in ESCC tumors was detected by Western blot. **L** Tumor image of the LEG110 tumor xenografts in the NC group and ASO treatment groups (7 per group). **M** Tumor volumes were measured and calculated in the NC and ASO treatment groups at specified times. **N** The weights of tumor xenografts were measured in the NC and ASO treatment groups. **O** The statistical analysis of IHC-positive staining of Ki67 in the NC and ASO treatment groups. **P** The protein expression of CERS6, RPN1, HSPA5, IRE1, and XBP1 in LEG110 tumor xenografts after ASO treatment targeting CERS6. *P < 0.05, **P < 0.01, ***P < 0.001.

Antisense oligonucleotides (ASOs) technology, as an emerging approach, has great potential for cancer treatment by targeting mRNA molecules to reduce the expression of specific proteins [42]. Owing to the high expression of CERS6 in ESCC and its influence on the development of ESCC, we investigated the potential of CERS6 as a therapeutic target through ASO treatment. Therefore, we designed two ASOs (ASO-1130, ASO-581) specifically targeting CERS6 and detected the mRNA and protein levels of CERS6 by qPCR and Western blot following ASO treatment. The protein (Fig. S6A, B) and mRNA (Fig. S6C, D) levels of CERS6 were significantly reduced in KYSE150 and KYSE450 cells. We then assessed the effect of ASOs targeting CERS6 on the proliferation of KYSE150 and KYSE450 cells by MTT and plate clone formation assays. Compared with the ASO negative control (NC) group, the proliferation of KYSE150 (Fig. 6A) and KYSE450 (Fig. 6B) cells was significantly inhibited after ASO treatment by MTT assays. In the plate clone formation assays, ASO treatment targeting CERS6 significantly decreased the cell clone formation ability of KYSE150 (Figs. 6C and S6E) and KYSE450 (Figs. 6D and S6F). These results suggested that ASO treatment targeting CERS6 could suppress the proliferation of ESCC cells. Moreover, we found that the protein levels of RPN1, HSPA5, IRE1, and XBP1 were significantly reduced in KYSE150 (Fig. 6E) and KYSE450 (Fig. 6F) cells after ASO treatment. In addition, we observed that ASO targeting CERS6 significantly increased ROS levels in KYSE150 (Fig. 6G) and KYSE450 (Fig. 6H) cells and promoted the apoptosis of KYSE150 (Fig. 6I) and KYSE450 (Fig. 6J) cells. The results show that ASO targeting CERS6 significantly inhibits the proliferation of ESCC cells in vitro.

Subsequently, we measured CERS6 protein levels in ESCC patient-derived xenografts (PDX) models to evaluate the effect of ASO-CERS6 treatment in vivo (Fig. 6K). The CERS6 protein levels were high in the case of LEG110, which was from a 69-year-old male patient diagnosed with moderately differentiated squamous cell (TNM stage IIIb). ASO NC or ASO-581 and ASO-1130 were administered intratumorally once every 5 days. Tumors were significantly reduced in size in the ASO-581 and ASO-1130 groups compared to the ASO NC group (Fig. 6L). The tumor volumes (Fig. 6M) and weights (Fig. 6N) were significantly reduced in the ASO-581 and ASO-1130 groups. Furthermore, the expression of Ki67 was reduced in the ASO-treated group (Figs. 6O and S6G), indicating that the proliferation ability was weakened after ASO treatment. Moreover, the protein levels of CERS6, RPN1, HSPA5, IRE1, and XBP1 were significantly reduced (Fig. 6P). The above data indicate that targeting CERS6 can impede tumor growth by blocking RPN1 degradation and the HSPA5-IRE1-XBP1 signaling pathway in ESCC in vivo.

## Discussion

CERS6 plays a crucial role in the progression of multiple types of cancer, including lung cancer, pancreatic cancer, gastric cancer, breast cancer, and ovarian cancer [21–23, 25]. Selective activation of ATF-6 in response to CERS6/C16 ceramide down-regulation protects head and neck squamous cell carcinoma cells from ER stress-induced apoptosis by preventing ER/Golgi [Ca²^+^] depletion and CHOP activation [39]. In this study, we found that CERS6 was significantly overexpressed in ESCC tissues relative to adjacent normal tissues and was associated with poor prognosis. CERS6 promoted the growth of ESCC in vitro and in vivo. Furthermore, CERS6 increased the stability of the RPN1 protein by directly binding with RPN1 to avoid its ubiquitination. Finally, an elevated CERS6 inhibited ROS activity through the HSPA5-IRE1-XBP1 signaling pathway in ESCC.

Astonishingly, our research revealed that CERS6 has a unique effect separate from ceramide synthase and is responsible for inhibiting apoptosis in ESCC. Although CERS6 did not exhibit canonical ceramide synthase activity, it was essential in protecting protein homeostasis through its TLC domain. CERS6 consists of two functional domains: the TLC domain and the Hox domain [43]. The TLC domain is predicted to contain five transmembrane α helices, and while its exact role is still unknown, it is associated with protecting protein homeostasis among other functions[44]. In our study, CERS6 could bind to RPN1 by its TLC domain, inhibit ubiquitination of RPN1 to increase its stability, and then promote the growth of ESCC. Our research supports the novel role of CERS6 in sustaining protein homeostasis in ESCC.

Many glycoproteins fail to fold when glycosylation is suppressed, accumulating defective proteins that induce an unfolded protein response (UPR) pathway in response to ER stress [45, 46]. Furthermore, interference with RPN1 induces UPR through the IRE1 signaling pathway [47]. In our study, we found that ER stress-related proteins, such as HSPA5, IRE1 and XBP1, decreased after CERS6 knockout. Moreover, the knockout of CERS6-induced ROS then significantly promoted the apoptosis of ESCC cells, which was reversible by rescuing RPN1. Studies have shown that endoplasmic reticulum stress-induced apoptosis inhibits the proliferation and invasion of breast cancer cells after RPN1 knockout [29]. Here, we have demonstrated that RPN1 plays a significant role in the progression of ESCC by enhancing the function of CERS6 through the HSPA5-IRE1-XBP1 signaling pathway. Therefore, the CERS6-RPN1 axis suppresses ROS and promotes ESCC cell proliferation. Our initial study explores a novel mechanism for understanding the oncogenesis of ESCC from the perspective of ROS and ER stress.

There is an urgent need to develop effective personalized molecular-targeted therapies for ESCC patients due to the lack of robust treatment efficacy [48, 49]. Here, we have discovered that CERS6 may be an important oncogenic factor in ESCC, making it a promising target for treating ESCC. However, CERS6 shares high structural similarity and conserved active sites with other family members, which presents significant challenges in developing selective small-molecule inhibitors [50]. Moreover, no effective inhibitors targeting CERS6 have been used in clinical trials yet.

To date, some pan-ceramide synthase inhibitors, such as fumonisin B1, have been reported and four functional inhibitors of CERSs have been derived from Fingolimod, however, they exhibit broad-spectrum activity and high toxicity, and lack selectivity toward CERS6 [51]. In contrast, ASOs exert their function through a clearly defined mechanism—recruiting RNase H to specifically degrade target mRNA, offering several advantages including precise targeting, rapid onset of action, and high specificity [42]. Moreover, previous studies have demonstrated that using ASO to target specific genes can hinder the progression of various tumors in mouse models, including hepatocellular carcinoma, colorectal, lung, and breast cancer [52, 53]. It is worth noting that several ASO treatments have been tested in clinical trials [54–56]. For instance, Custirsen (OGX-011), a second-generation ASO, has been shown to inhibit tumor growth in lung cancer models and is currently in phase III clinical trials (NCT01630733) [55]. In our study, we designed an ASO treatment targeting CERS6 and proved that it significantly reduced the mRNA and protein levels of CERS6 and inhibited ESCC cell proliferation in vitro and in vivo. Moreover, the ASO treatment promoted the production of ROS and induced apoptosis by down-regulating the RPN1-HSPA5-IRE1-XBP1 signaling pathway. Therefore, our research has provided a promising target and treatment option focusing on CERS6 for ESCC patients.

## Conclusion

In ESCC, CERS6 was highly expressed and protected the RPN1 protein by inhibiting its ubiquitination. The increased RPN1 enhanced the HSPA5/IRE1/XBP1 signaling pathway and decreased the ER stress and ROS levels, resulting in the promotion of cell proliferation. Moreover, targeting CERS6 inhibited the growth of ESCC through the RPN1-IRE1-XBP1 signaling pathway, indicating that CERS6 may be a promising therapeutic target for ESCC.

## Materials and methods

### Reagents and antibodies

In our research, we employed the Annexin V-FITC/PI apoptosis kit (AT101C-100, Multi Sciences) and reactive oxygen species assay kit (S0033S, Beyotime Biotechnology). The following antibodies were used in this study: anti-CERS6 (ab115539, Abcam); anti-RPN1 (12894-1-AP, Proteintech); anti-HSPA5 (sc-13539, Santa Cruz Biotechnology); anti-IRE1 (sc-390960, Santa Cruz Biotechnology); anti-XBP1 (sc-8015, Santa Cruz Biotechnology); anti-Myc (sc-40, Santa Cruz Biotechnology); anti-Ki67 (ab15580, Abcam); anti-GAPDH (AB-M-M001, Good Here); anti-Tubulin (EM0103, Huabio); anti-V5-Tag (M167-3, Medical Biological Laboratories) and anti-Flag-Tag (M185-3L, Medical Biological Laboratories).

### Cell culture

The human ESCC cell lines KYSE30, KYSE70, KYSE140, KYSE150, KYSE410, KYSE450, and KYSE510 were purchased from the Cell Bank of the Chinese Academy of Sciences (Shanghai, China). The Shantou human embryonic esophageal epithelial (SHEE) cell line, a typical human esophagus immortalized epithelial cell line, was provided by Professor Enmin Li. These cells were cultured in RPMI-1640 medium supplemented with 10% fetal bovine serum (FBS), 0.1 g/mL streptomycin, and 100 U/mL penicillin (Sigma, USA). The cells were incubated in a humidified incubator at 37 °C with 5% CO_2_. All cells were verified to be mycoplasma-free and analyzed by using STR analysis.

### Tissue microarray

Tissue microarray, including 59 ESCC and 60 adjacent normal tissues, was obtained from the Pathology Department of Henan Cancer Hospital after obtaining informed consent from patients. The expression of CERS6 was assessed by immunohistochemical staining based on the percentage of DAB-staining-positive cells as previously described [45, 57]. Kaplan and Meier’s survival analysis evaluated the relationship between CERS6 expression and patient survival. Moreover, the data on characteristics of CERS6 in ESCC patients were analyzed using GraphPad Prism 7.0 software by Chi-square statistics, and the standard deviation was added.

### Immunohistochemistry (IHC)

As in our previous studies, we followed the protocol and scoring methods accordingly [45, 57]. Tissue slides were baked at 65 °C for 2 h, then dewaxed in xylene and rehydrated through a graded ethanol series. After rinsing in 1×TBST, antigen retrieval was performed using an acidic buffer in a microwave for 10 min. Endogenous peroxidase activity was blocked with 3% H₂O₂ for 10 min. Slides were incubated overnight with primary antibody at 4 °C, followed by horseradish peroxidase (HRP)-conjugated secondary antibody at 37 °C for 30 min. Immunoreactivity was visualized using 3,3′-Diaminobenzidine (DAB), and slides were counterstained with hematoxylin. Differentiation was performed with 0.1% hydrochloric acid alcohol, followed by a 30-min rinse in tap water. Finally, slides were dehydrated and mounted with neutral resin.

CERS6 expression in tissue arrays was assessed using a semi-quantitative immunoreactivity scoring system. Slides were scanned using the TissueFAXS PLUS system (TissueGnostics, Vienna, Austria) and analyzed using HistoQuest software. Regions of ESCC and adjacent tissue were manually selected. DAB and hematoxylin signals were quantified via automated nuclear and compartment segmentation. The percentage of CERS6-positive cells was calculated for each sample. Patients were divided into high and low expression groups based on the median CERS6-positive cell percentage.

### Western blot

Cells were lysed in RIPA buffer (50 mM Tris-HCl, pH 7.4, 150 mM NaCl, 1% NP-40, 0.5% sodium deoxycholate, 0.1% SDS) supplemented with protease and phosphatase inhibitors. Lysates were incubated on ice for 30 min and centrifuged at 12,000 rpm for 30 min at 4 °C. Protein concentrations were measured using a BCA assay. Samples (1 μg/μL) were boiled at 100 °C for 10 min, separated by SDS-PAGE, and transferred to PVDF membranes. Membranes were blocked with 5% non-fat milk for 1 h at room temperature (RT), incubated with primary antibodies overnight at 4 °C, and then with secondary antibodies for 2 h at RT. Bands were visualized using an ECL system and captured with an Image Reader. Each experiment was independently repeated three times.

### Patients’ tissues

Tissue samples (normal [N], peri-tumor [P], and cancerous [C] tissues) were collected from 21 ESCC patients at Linzhou Tumor Hospital (Henan, China) with informed consent. CERS6 protein levels were assessed by Western blot. Band intensities were quantified using ImageJ by converting images to grayscale and calculating gray values.

### CRISPR/Cas9-knockout, lentivirus infection and transfection

The primers to knock out target genes were designed and synthesized by Shengong Bioengineering Co., Ltd. Double-stranded DNA was annealed at 100 °C and slowly lowered to RT. The vectors of Lenti-CRISPR V2 were connected by enzyme digestion to get the target plasmids. The human CERS6 mRNA and protein sequences were retrieved from NCBI RefSeq (accession NM_001256126.2, NP_001243055.1). The primers for sgCECR6 and sgRPN1 are shown in Table S1. The above target plasmids were transfected into stable and active 293T cells, and the virus supernatant was collected 24 h and 48 h after the new medium was replaced. The liquid was filtered through a 0.22-µm filter and stored for later use. KYSE150 and KYSE450 cells were infected with the viruses by adding polybrene (8 μL/10 mL). Cells were screened with puromycin (2 μg/mL) 3 days after infection. The effectiveness of gene knockout was verified by Western blot analysis.

### Limiting dilution cloning (LDC)

The cells were harvested through trypsinization and counted. Afterward, they were diluted to 2000 cells per 50 mL. We seeded 200 µL of the diluted cells into the first 96-well plate while the remaining cells were filled with medium to a total volume of 50 mL. Similarly, 200 µL of the diluted cells were transferred to another 96-well plate and repeated five times. The well containing a single cell was marked. For single cloning and knockout verification, each clone was subjected to genomic DNA extraction using a solution of 50 mM NaOH and 1 M Tris (pH 7.0), followed by purification with phenol and chloroform. PCR products were amplified using Q5 high-fidelity polymerase. For the CERS6 gene, the primers are shown in Table S2. The PCR products were visualized on 2.0% gels and purified for sequencing.

### MTT assay

2.5 × 10^3^ KYSE70, 3 × 10^3^ KYSE150 and 3 × 10^3^ KYSE450 cells were seeded in 96-well plates. The cells were incubated for 0 h, 24 h, 48 h, 72 h, and 96 h, and then 20 μL of [3-(4,5)-dimethylthiazol-2-yl]-2,5-diphenyltetrazolium bromide (MTT) solution (5 mg/mL) was added to each well, followed by an additional 2 h incubation at 37 °C. After incubation, the MTT solution was removed and replaced with 100 μL dimethyl sulfoxide (DMSO) in each well to dissolve the formazan crystals that developed in the viable cells. To assess cell viability, the absorbance at 490 nm was measured. The three replicates were guaranteed in every experiment, and every experiment was independently repeated three times.

### Plate clone formation assay

Three hundred KYSE cells were seeded into each well of 6-well plates and then cultured at 37 °C and 5% CO_2_ for 10–12 days. After the incubation, the colonies were washed twice with cold phosphate-buffered saline (PBS) and fixed with 4% paraformaldehyde for 30 min. Subsequently, 0.5% crystal violet was applied to stain the colonies for 5–10 min. The number of clones was calculated and examined by capturing photographs. Each experimental group had 3 parallel wells, independently repeated three times.

### Soft agar assay

ESCC cells were utilized with 8 × 10^3^ per well and suspended in a 0.3% agar solution mixed with 1× BME medium (1% L-glutamine, 10% FBS, and 0.1% gentamicin). We then layered the cell suspension onto a 6-well plate with a base layer of 0.6% agar in 1× BME medium. The cells were incubated for 2 weeks. Finally, the colonies were photographed and counted using the IN-Cell Analyzer 6000 [58]. Each experimental group had 3 parallel wells, and they were independently repeated three times.

### Cell-derived xenograft model

One hundred microliters of cells (KYSE150: 5 × 10^7^ cells/mL, KYSE450: 7 × 10^7^ cells/mL) were subcutaneously injected into each mouse. On day 7 post-inoculation, tumor size was measured with a vernier caliper. The mice were randomly divided into 3 groups, with 6 female CB17/SCID mice of 5–6 weeks in each group. The tumor volumes of the mice were monitored every 3 days and calculated using the formula: volume = (*a* × *b*^2^)/2, where ‘*a*’ was the longer diameter and ‘*b*’ was the shorter diameter. When the tumor was about 400 mm^3^ in the sgScramble group, mice were euthanized, and tumors were harvested, weighed, and photographed. All animal experiments were performed in compliance with animal ethical standards.

### The pull-down assay combined with mass spectrometry analysis

After transfecting the PLVX-IRES-Puro-3×Flag-CERS6 plasmid into 293F cells, the recombinant CERS6 protein was purified using M2 beads. Then, the CERS6 protein was incubated with KYSE150 and KYSE450 cell lysates overnight at 4 °C. Next, the beads were washed five times and boiled at 100 °C for 30 min. Subsequently, the samples were electrophoresed with 8% SDS-PAGE gel and dyed with coomassie blue for 30 min. After that, the samples were washed in a shake overnight for decolorization. The gel pieces were decolorized by incubating them with a decolorization solution at 37 °C until the gel shrank to white. The gel pieces were finely cut and washed twice for enzymatic hydrolysis. Acetonitrile was added to the system, vortexed, and incubated at RT until the glue became transparent. The gel was concentrated in SpeedVac for 5 min and digested by enzymatic hydrolysis. Finally, the peptides were analyzed using Nano-LC–MS/MS (AB SCIEX TripleTOF 5600, Framingham, MA, USA).

### Cell immunofluorescence assay

Cells were seeded on coverslips in 24-well plates and fixed with 4% paraformaldehyde for 20 min at ~80% confluence, followed by permeabilization with 0.5% Triton X-100 for 10 min. After blocking with 1% BSA-PBST for 1 h, coverslips were incubated with primary antibodies (1:50 in 3% BSA-PBST) overnight at 4 °C in a dark humid chamber. The next day, slides were washed (3×, 3 min each) with 1% BSA-PBST, incubated with fluorophore-conjugated secondary antibodies for 2 h, and stained with DAPI for 5 min. Anti-fade mounting medium was applied, and images were captured using a fluorescence microscope (SOPTOP IRX50, Sunny Optical, China). Each group included three replicates, and experiments were repeated independently three times.

### Co-immunoprecipitation assay

2 × 10^7^ ESCC cells were lysed in 300 μL buffer (50 mM Tris pH 8.0, 150 mM NaCl, 0.5% NP-40) at 4 °C, and protein concentration was determined using the BCA assay. The lysate was adjusted to 1 mg/mL, pre-cleared with 20 μL protein A/G beads at 4 °C for 2 h, and centrifuged (3000 rpm, 5 min). Supernatants were incubated overnight at 4 °C with 2 μg CERS6 antibody and 20 μL protein A/G agarose beads. The next day, pellets were washed 5 times with lysis buffer, and bound proteins were eluted with 2×loading buffer, boiled at 100 °C for 10 min, and analyzed by Western blot.

### Computational docking model

To predict the potential interaction between CERS6 and RPN1, we performed molecular docking using the HDOCK server (http://hdock.phys.hust.edu.cn/) [59–63], which combines template-based modeling with ab initio docking algorithms. AlphaFold-predicted structures of CERS6 (AF-Q6ZMG9-F1) and RPN1 (AF-P04843-F1) were downloaded from the PDB website (https://www.rcsb.org/) and used as input. Based on the docking score, the docking model was selected for analysis and visualization using PyMOL (Schrödinger, LLC).

### RNA extraction

1 × 10⁶ to 1 × 10⁷ ESCC cells were lysed in 1 mL of TRIzol reagent and incubated at RT for 3 min. Subsequently, 200 μL chloroform was added to each tube, and the tubes were shaken 6–8 times at RT for 3 min. Then, the samples were centrifuged at 4 °C, 13000 rpm for 10 min and divided into three layers. The top layer (water phase) was drained into the EP tube. An equal volume of isopropyl alcohol was added to the EP tube, thoroughly mixed, and left at RT for 4 min. The EP tubes were centrifuged at 13,000 rpm for 20 min at 4 °C, and the supernatant was collected. Afterward, 1 mL freshly prepared 75% ethanol was added to each RNA-containing tube, and the EP tubes were gently shaken. Then, this system was centrifuged at 13,000 rpm for 5 min at 4 °C. Finally, RNA precipitation was dissolved in enzyme-free sterile water.

### Quantitative real-time PCR (qPCR)

The total RNA was extracted from the cells using the TRIzol reagent. Then, we followed the manufacturer’s instructions and performed reverse transcription using the PrimeScript RT-PCR kit (RR90A, Takara). The expression of CERS6 mRNA was analyzed using SYBR Premix Ex Taq (RR420A, Takara). Quantitative analysis was performed using the 2 ^−ΔΔ^Ct method. The sequences for qPCR are shown in Table S3. The three replicates were guaranteed in every experiment, and every experiment was independently repeated three times.

### Cycloheximide (CHX) chase assay

The ESCC cells were seeded in 10 cm dishes to achieve a 50% confluence. Cells were then treated with 50 μg/mL CHX. Two time points, 0 h and 8 h, were selected for each experimental group. Cells were collected and lysed once reaching the corresponding time point, and protein concentration was determined using the BCA method. The degradation rate of RPN1 was assessed using Western blot. Every experiment was independently repeated three times.

### Ubiquitination assay

The corresponding plasmids (pLVX-IRES-Puro-3×Flag-CERS6, pcDNA3.1-V5-RPN1, pLVX-IRES-Puro-3×Flag-ASB11, and Myc-tagged ubiquitin expression plasmid) were transfected into 293T cells. After the cells were scraped and the concentration of lysed cells was calculated, we employed a Western blot to determine the transfer efficiency. Afterward, 500 μg protein was incubated with 20 μL A/G agarose beads on a rotating shaker at 4 °C for 2 h. The mixture was centrifuged at 3000 rpm for 5 min, and the supernatant was mixed with 20 μL A/G agarose beads and the corresponding antibody. The mixture was incubated overnight on a rotary shaker at 4 °C. The next day, the supernatant was discarded after centrifugation at 3000 rpm for 5 min. The pellet was washed 5 times with 1 mL of lysate on a rotating shaker at 4 °C, followed by centrifugation at 3000 rpm for 5 min after each wash to remove the supernatant. Finally, the sediment in 20 μL 2×loading buffer was boiled for 10 min at 100 °C, and the result was detected by Western blot. Every experiment was independently repeated three times.

### ROS assay

We digested and spread 1.0 × 10^5^ KYSE150 and KYSE450 cells into 6 cm dishes and then cultured them overnight in the incubator. The next day, the cells were taken out and rinsed twice with PBS. The culture medium was then replaced with a working solution of 2′,7′ –dichlorofluorescin diacetate (DCFH-DA), which came from the reactive oxygen species assay kit (S0033S, Beyotime Biotechnology). The cells were returned to the incubator and incubated for 30 min. After incubation, the cells were washed twice with PBS. The cells were digested and centrifuged at 1000 rpm for 3 mins. The supernatant was discarded, and the cells were thoroughly mixed with 1 mL of PBS. The fluorescence intensity of DCFH-DA was measured and analyzed using flow cytometry. The three replicates were guaranteed in every experiment, and every experiment was independently repeated three times.

### Cell apoptosis assay

1.0 × 10^5^ ESCC cells were spread in 6 cm dishes and incubated for 72 h. The cells were digested and centrifuged at 4 °C at 1000 rpm for 3 min. Then, the cells were rinsed twice with chilled PBS. The Annexin V-FITC/PI apoptosis kit (AT101C-100, Multi Sciences) was employed according to the protocol. 0.5 mL of the diluted buffer was mixed into each sample. Then, 10 μL PI and 5 μL Annexin V-FITC solution were added. The mixtures were thoroughly incorporated and incubated on ice in the dark for 3–5 min. FITC and PI fluorescence was detected and analyzed by flow cytometry. The three replicates were guaranteed in every experiment, and every experiment was independently repeated three times.

### Antisense oligonucleotide treatment

Two ASOs targeting CERS6 were designed and synthesized by Sangon Biotech (Shanghai) Co., Ltd. The ASO concentrations for cells were 10 nM and 20 nM. For the in vivo study, the ASOs were injected directly into the tumors (5 nmol/mouse/every time) every 5 days when the xenograft volume reached about 100 mm^3^. Tumor volumes were monitored every 3 days and calculated as 0.5 × length × width^2^. The sequences for ASO are shown in Table S4.

### Patient-derived xenograft model

Female CB17/SCID mice aged 5–6 weeks were bought from Cyagen Biosciences, Inc. (Suzhou, China) and were housed in a specialized pathogen-free (SPF) barrier facility. The facility maintained a temperature of 20 ± 2 °C, 50 ± 10% relative humidity, and a 12-h light/dark cycle. The mice were provided with unlimited access to food and water. LEG110 PDX tumor tissues were fragmented and implanted into the back of each mouse. The mice were randomly divided into 3 groups, with 7 in each group: the vehicle group, the CERS6-ASO-581 group, and the CERS6-ASO-1130 group. Tumor volume and body weight were monitored every 3 days. When the tumors in the vehicle group reached an average volume of about 1000 mm^3^, all xenografts in the three groups were extracted after euthanizing the mice [18]. We randomly selected three tissues from each group for multiple comparisons in the animal experiments. We then used these samples to verify the protein levels of CERS6, RPN1, HSPA5, IRE1, and XBP1 using Western blot. The animal experiments in this study were conducted with the approval of the Research Ethics Committee of Zhengzhou University (ZZUIRB 2022-70).

### Statistical analysis

For the experiments, including MTT assay, plate clone formation assay, soft agar assay, cell immunofluorescence assay, qPCR, ROS assay, and cell apoptosis assay, three replicates were performed, and every experiment was independently repeated three times. For Western blot, Co-IP assay, and CHX chase assay, every experiment was independently repeated three times. The experimental data are presented as means ± standard deviation (SD) unless stated otherwise. Statistical analysis was performed using GraphPad Prism Version 7.0 or SPSS 21.0 software. First, the normality and homogeneity tests of variance were conducted to ensure the validity of the data. If both tests were passed, a one-way ANOVA was used. If the data met the homogeneity of variance assumption, the least significant difference method was used. Moreover, a non-parametric test was used if the variance was inconsistent. Pearson’s correlation analyses were conducted to determine the expression and correlation between the two groups. The graphs were produced by R/ggstatsplot, which was downloaded from https://www.aclbi.com/static/index. html. Differences were considered statistically significant at *P* < 0.05.

## Supplementary information

Supplementary Figure Lengends and Tables

Supplementary Figures

Original Western blots

## Data Availability

All data and materials related to the results of this study are available in the manuscript and the supplementary materials.
