# Supplementary material for: CERS6 promotes esophageal squamous cell carcinoma proliferation by increasing the stability of RPN1
Source: Cell Death Discov. 2025 Nov 7;11:512. doi: 10.1038/s41420-025-02727-y (PMC12594893; doi:10.1038/s41420-025-02727-y)
Supplement: Supplementary file 1 — Supplementary Figure Lengends and Tables [file 41420_2025_2727_MOESM1_ESM.pdf]

CERS6 promotes esophageal squamous cell carcinoma proliferation by increasing the stability of RPN1

Wenjing Chen[1,3,4 †], Yuxin Zhai[1,2,4 †], Xiaoxiao Yang[1,3,4 †] , Weizhe Zhang[1,3,4], Dan Gao[1,3,4], Xiaokun Zhao[1,3,4], Nannan Zhao[6], Yuhan Zhang[1,3,4], Qiang Yuan[1,3,4], Zhiying Zhao[1], Qiong Wu[1,2,3,4], Yifei Xie[1], Jimin Zhao[1,5], Zigang Dong[1,2,3,4 *], Kangdong Liu[1,2,3,4,5*], Yanan Jiang[1,2,3,4,5*]

[1]State Key Laboratory of Metabolic Dysregulation & the Prevention and Treatment of Esophageal Cancer, School of Basic Medical Sciences, Zhengzhou University, Zhengzhou, Henan, 450052, China

[2]Tianjian Laboratory of Advanced Biomedical Sciences, Zhengzhou 450000, Henan, China

[3]China-US (Henan) Hormel Cancer Institute, Zhengzhou 450000, Henan, China

[4]Department of Pathophysiology, School of Basic Medical Sciences, Zhengzhou University, Zhengzhou 450000, Henan, China

[5]Henan International Joint Laboratory of Cancer Chemoprevention, Zhengzhou University, Zhengzhou 450000, Henan, China

[6]Department of Neurosurgery, The First Affiliated Hospital of Zhengzhou University, Zhengzhou 450000, Henan, China

Supplementary Figure Lengends

Fig. S1 CERS6 is overexpressed in EC

A Protein levels of CERS6 in ESCC tissues based on clinical stages. **B** The mRNA levels of CERS6 in esophageal cancer from TCGA database based on clinical stages. TPM, transcription per million. **C** The mRNA levels of CERS6 in different cancers from the TCGA and GTEx database. **D** The CERS6 protein expression in 6 paired normal (N), peri-tumor (P), and cancerous tissues (C) of ESCC patients by Western blot. **P* < 0.05, ***P* < 0.01, ****P* < 0.001.

Fig. S2 CERS6 promotes cell proliferation of ESCC *in vitro* and *in vivo*

A, B Western blot was used to detect the knockout efficiency of CERS6 in KYSE150 (**A**) and KYSE450 (**B**) cells. **C, D** The rescue efficiency of CERS6 protein levels in KYSE150 (**C**) and KYSE450 (**D**) sgCERS6 cells.

Fig. S3 CERS6 binds with RPN1 in ESCC cells

A The gel picture for MS. **B** CERS6 protein levels in EC and adjacent tissues were assessed by proteomic data from 124 patients with clinical information. **C** Kaplan-Meier analysis of SEC22B by proteomic data from 124 patients with clinical information. **D** Relationship diagram between CERS6 and SEC22B in the Clinical Health Trust Home database (https://www.aclbi.com/).

Fig.S4 CERS6 promotes cell proliferation by stabilizing the RPN1 protein in ESCC

A,B The protein expression of RPN1 in RPN1 knockout KYSE150 (**A**) and KYSE450 (**B**) cells was detected by Western blot. **C,D** Cell viability of stable knockout RPN1 in KYSE150 (**C**) and KYSE450 (**D**) cells was measured by MTT assay.

Fig.S5 The CERS6-RPN1 axis inhibits the ROS-mediated apoptosis by the HSPA5-IRE1-XBP1 signaling pathway in ESCC

A,B Representative images of the ROS levels in KYSE150 (**A**) and KYSE 450 (**B**) sgScramble, sgCERS6-2, sgCERS6-5 cells. **C,D** Representative images of the apoptosis assay in KYSE150 (**C**) and KYSE450 (**D**) sgScramble, sgCERS6-2, sgCERS6-5 cells. **E, F** Representative images of the ROS levels in rescuing RPN1

after knockout CERS6 in KYSE150 (**E**) and KYSE450 (**F**) cells. **G, H** Representative images of the apoptosis assay after rescuing RPN1 in knockout CERS6 KYSE150 (**G**) and KYSE450 (**H**) cells.

Fig. S6 ASO treatment targeting CERS6 inhibits the cell proliferation of ESCC *in vitro* and *in vivo*

A,B Western blot of CERS6 protein in KYSE150 (**A**) and KYSE450 (**B**) cells after ASO treatment. **C,D** The mRNA level of CERS6 was detected after ASO treatment in KYSE150 (**C**) and KYSE450 (**D**) cells by qPCR. **E,F** The representative plate clone images after ASO treatment for CERS6 in KYSE150 (**E**) and KYSE450 (**F**) cells were obtained by plate clone formation assay. **G** Representative immunohistochemical images of Ki67 in LEG110 tumor xenografts of the NC and ASO treatment groups. Scale bar: 50μm.

Supplementary Table 1 The sequence of sgRNA primers

Gene（Human）	Sequence
sgCERS6-2-F	5'- CACCGATGTTGAGGGCTATGGCGCA-3'
sgCERS6-2-R	5'- AAACTGCGCCATAGCCCTCAACATC-3'
sgCERS6-5-F	5'- CACCGCCTCGTATTCCACAACCAG-3'
sgCERS6-5-R	5'- AAACCTGGTTGTGGAATACGAGGC-3'
sgRPN1-3-F	5'- CACCGTGGAGAGCTACACCAAGCTG -3'
sgRPN1-3-R	5'- AAACCAGCTTGGTGTAGCTCTCCAC -3'
sgRPN1-4-F	5'- CACCGTACCAGAGACAGCCAGATAG -3'
sgRPN1-4-R	5'- AAACCTATCTGGCTGTCTCTGGTAC -3'

Supplementary Table 2 The primer sequences for LDC

Gene（Human）	Sequence
sgCERS6-2-F	5'- CTCGGGGCCAGCCGGGCGCGC-3'
sgCERS6-2-R	5'- TAGTTGTACCAGCAATGCCTC-3'
sgCERS6-5-F	5'- GATTTGTAGCCAAACCGTGCG-3'
sgCERS6-5-R	5'- ACAGGAGATCACACATTTTCT-3'

Supplementary Table 3 The primer sequences for qPCR

Gene（Human）	Sequence
CERS6-F	5'-GACGCAATCAGGAGAAGCCAAG-3'
CERS6-R	5'-GGTAGTTGTACCAGCAATGCCTC-3'
RPN1-F	5'-CTGACTGTGAAGATCATCCTGCC-3'
RPN1-R	5'-GTCCAGATAGGTGTAGTGCAGC-3'
β-actin-F	5'-CACCATTGGCAATGAGCGGTTC-3'
β-actin-R	5'-AGGTCTTTGCGGATGTCCACGT-3'

Supplementary Table 4 The gene sequences for ASO

Gene（Human）	secquence(5′- 3′)
CERS6-ASO-581	(lA)*(lA)*(lT)*(dG)*(dA)*(dA)*(dA)*(dA)*(dT)*(dC)*(dT)*(dC)*(dC)*(dA)*(dC)*(dA)*(lT)*(lG)*(lC)
CERS6-ASO-1130	(lT)*(lT)*(lC)*(dA)*(dC)*(dA)*(dA)*(dT)*(dC)*(dA)*(dA)*(dG)*(dT)*(dA)*(dA)*(dG)*(lA)*(lC)*(lC)
