# Supplementary material for: CERS6 promotes esophageal squamous cell carcinoma proliferation by increasing the stability of RPN1
Source: Cell Death Discov. 2025 Nov 7;11:512. doi: 10.1038/s41420-025-02727-y (PMC12594893; doi:10.1038/s41420-025-02727-y)

Supplementary Figure 1

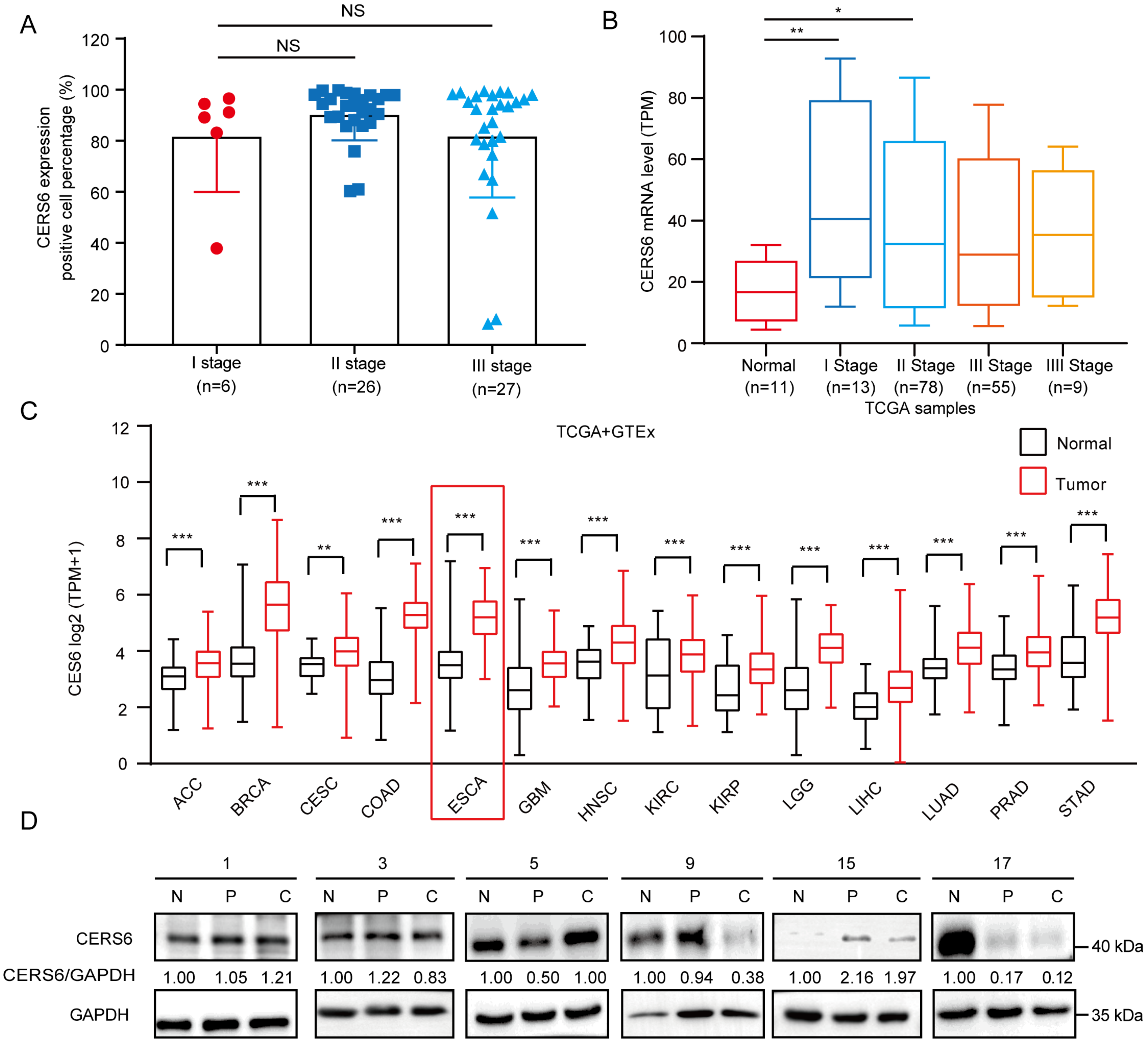

Supplementary Figure 2

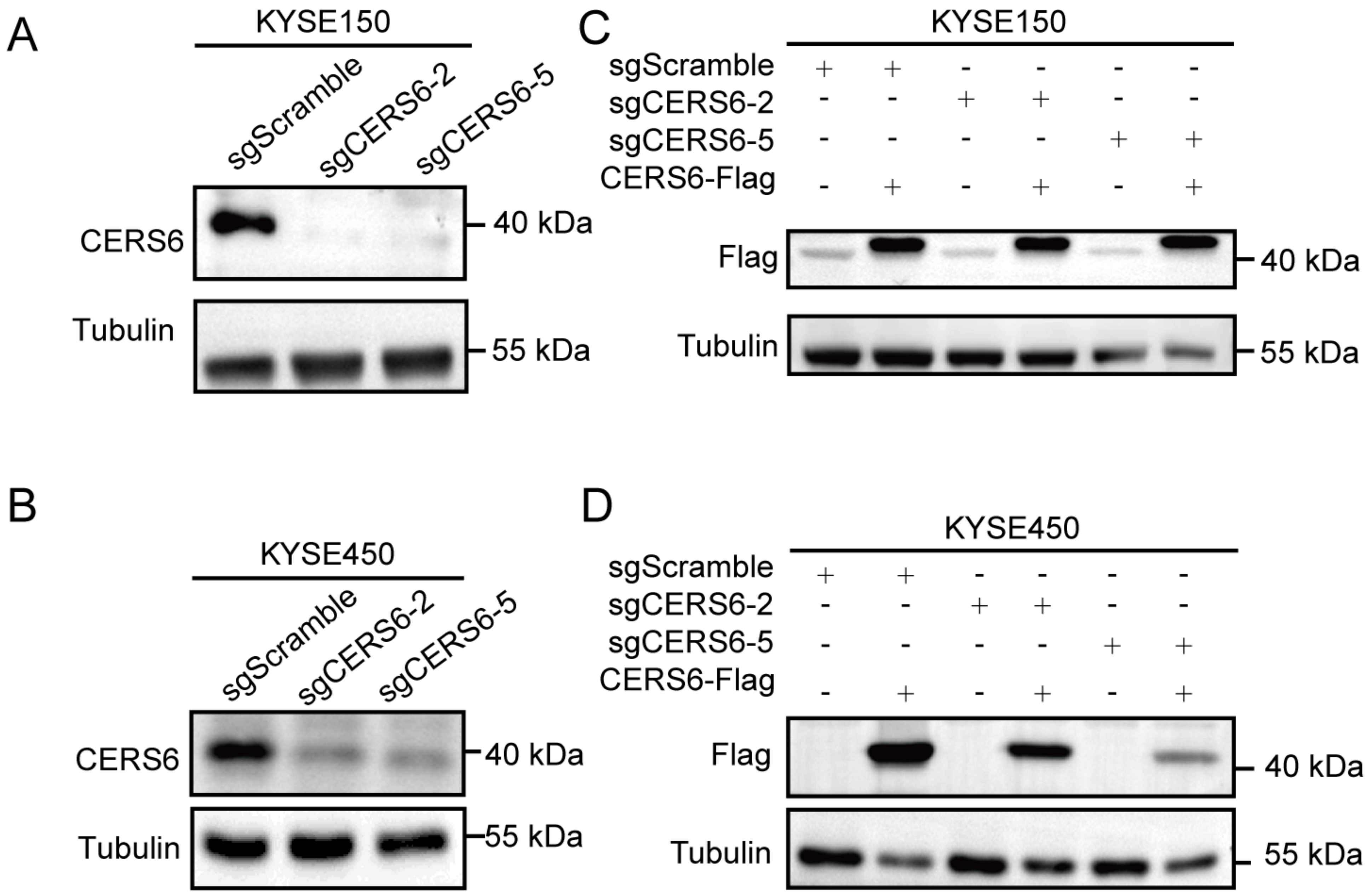

Supplementary Figure 3

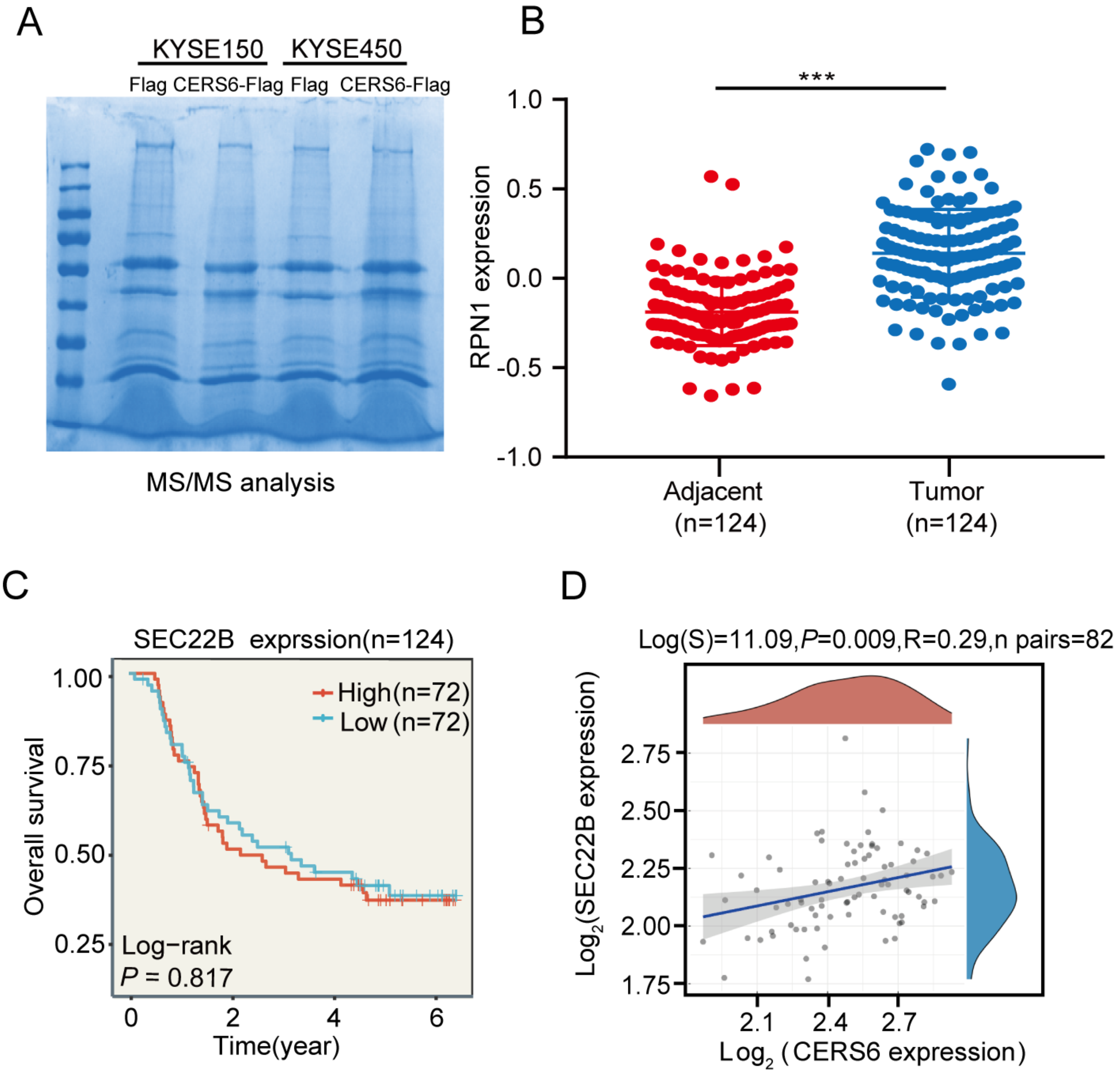

Supplementary Figure 4

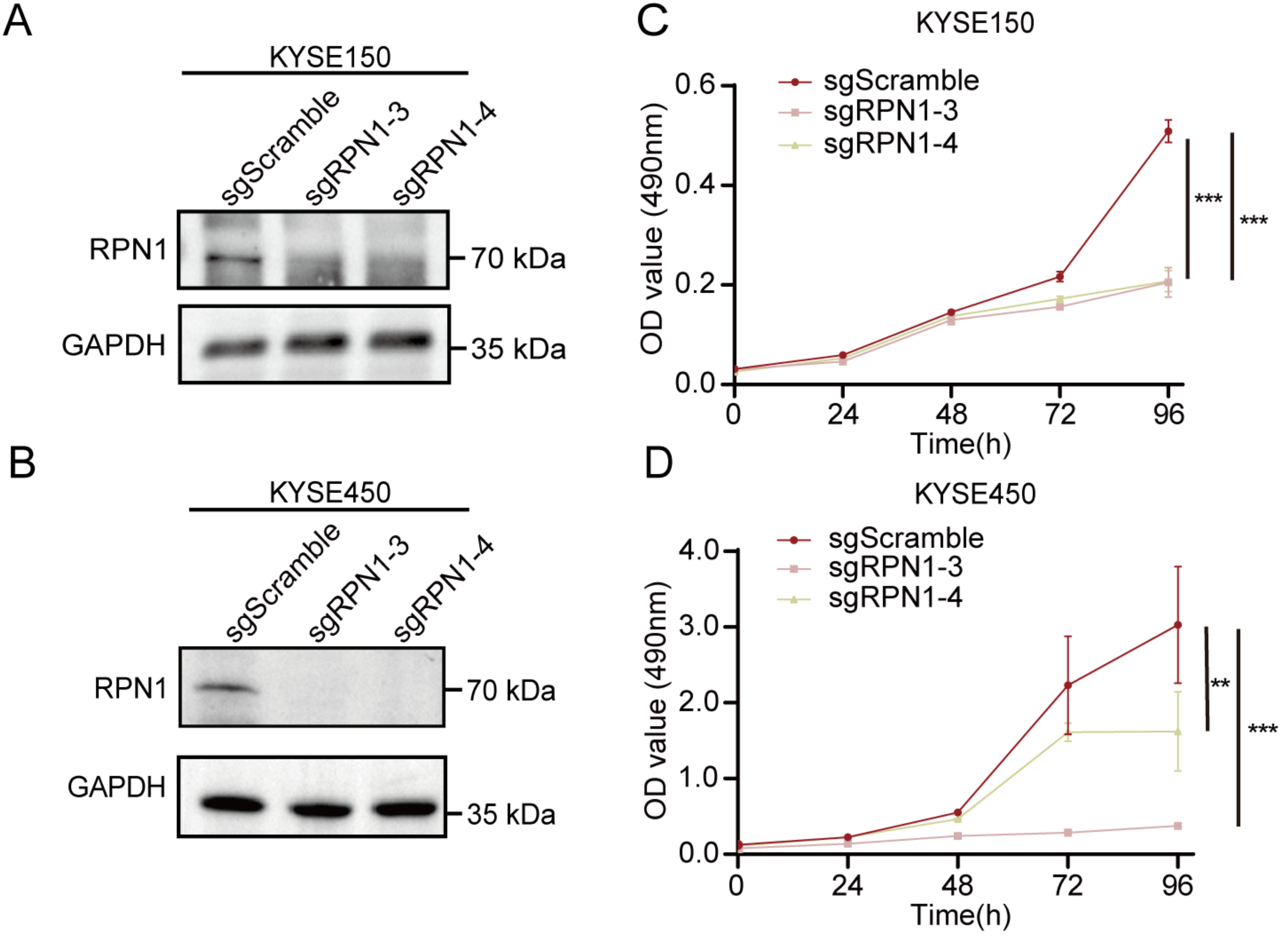

Supplementary Figure 5

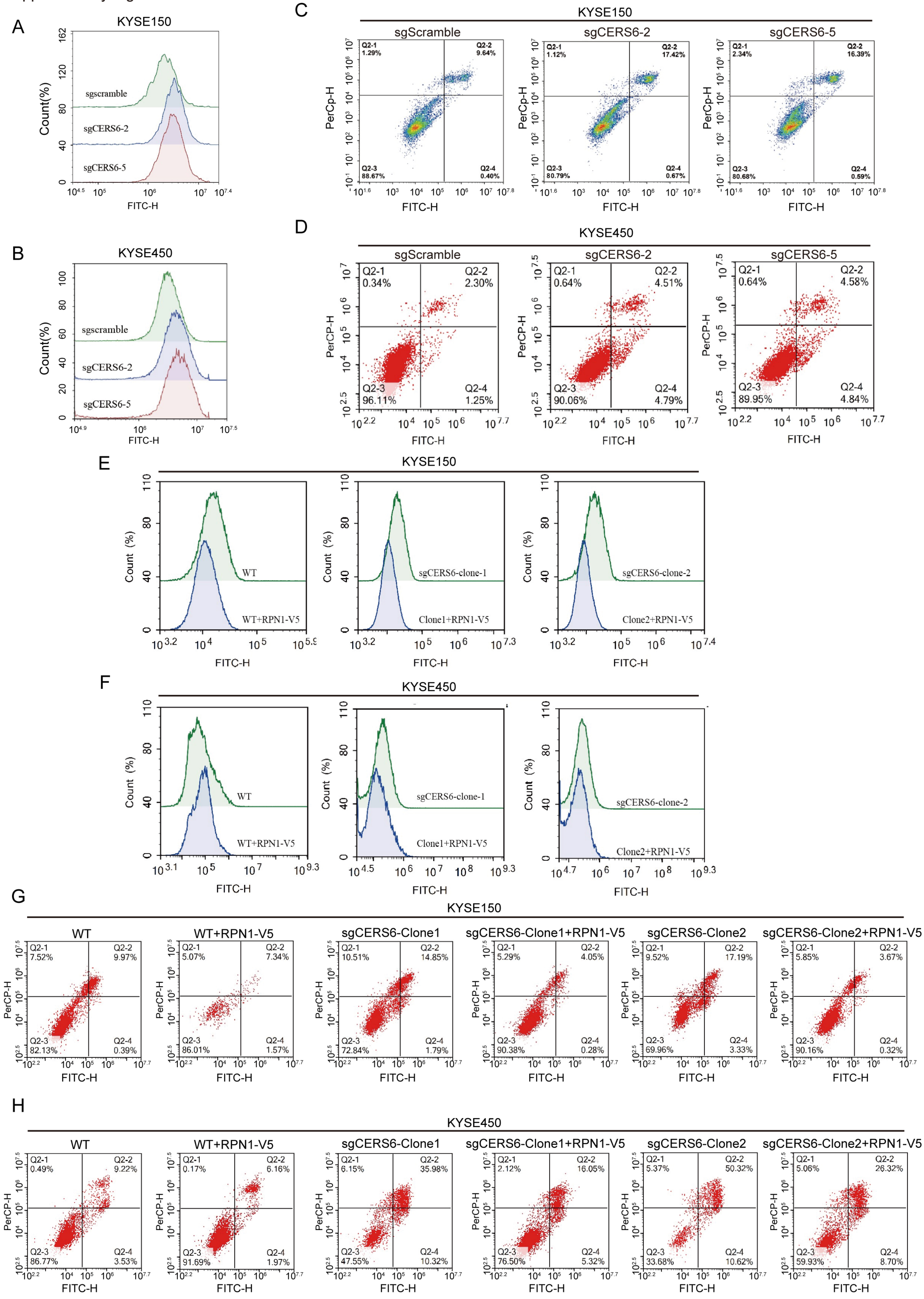

Supplementary Figure 6

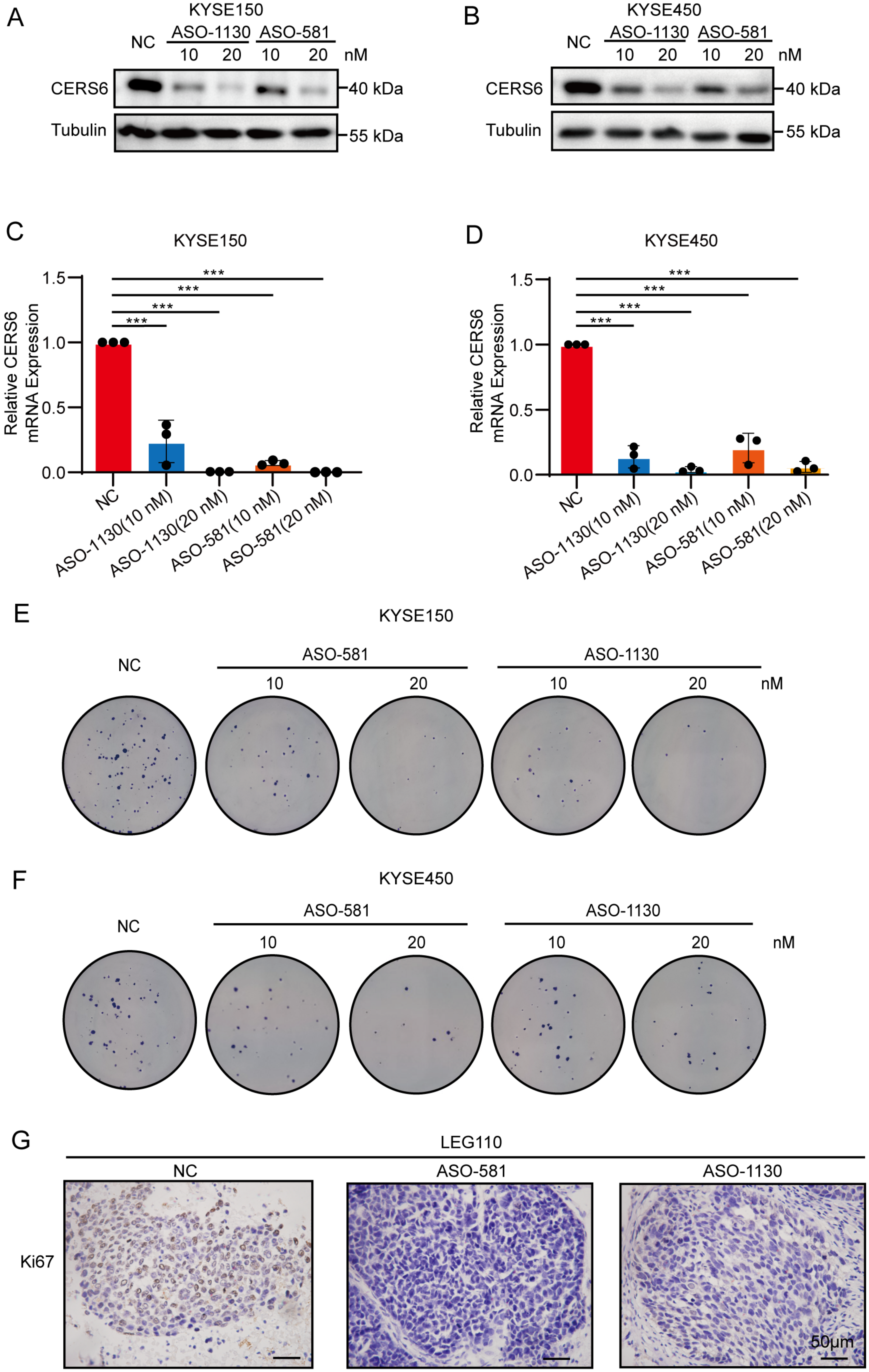

Supplement: Supplementary file 2 — Supplementary Figures [file 41420_2025_2727_MOESM2_ESM.pdf]
