# Supplementary material for: CERS6 promotes esophageal squamous cell carcinoma proliferation by increasing the stability of RPN1
Source: Cell Death Discov. 2025 Nov 7;11:512. doi: 10.1038/s41420-025-02727-y (PMC12594893; doi:10.1038/s41420-025-02727-y)

# Figure 1 Western blot

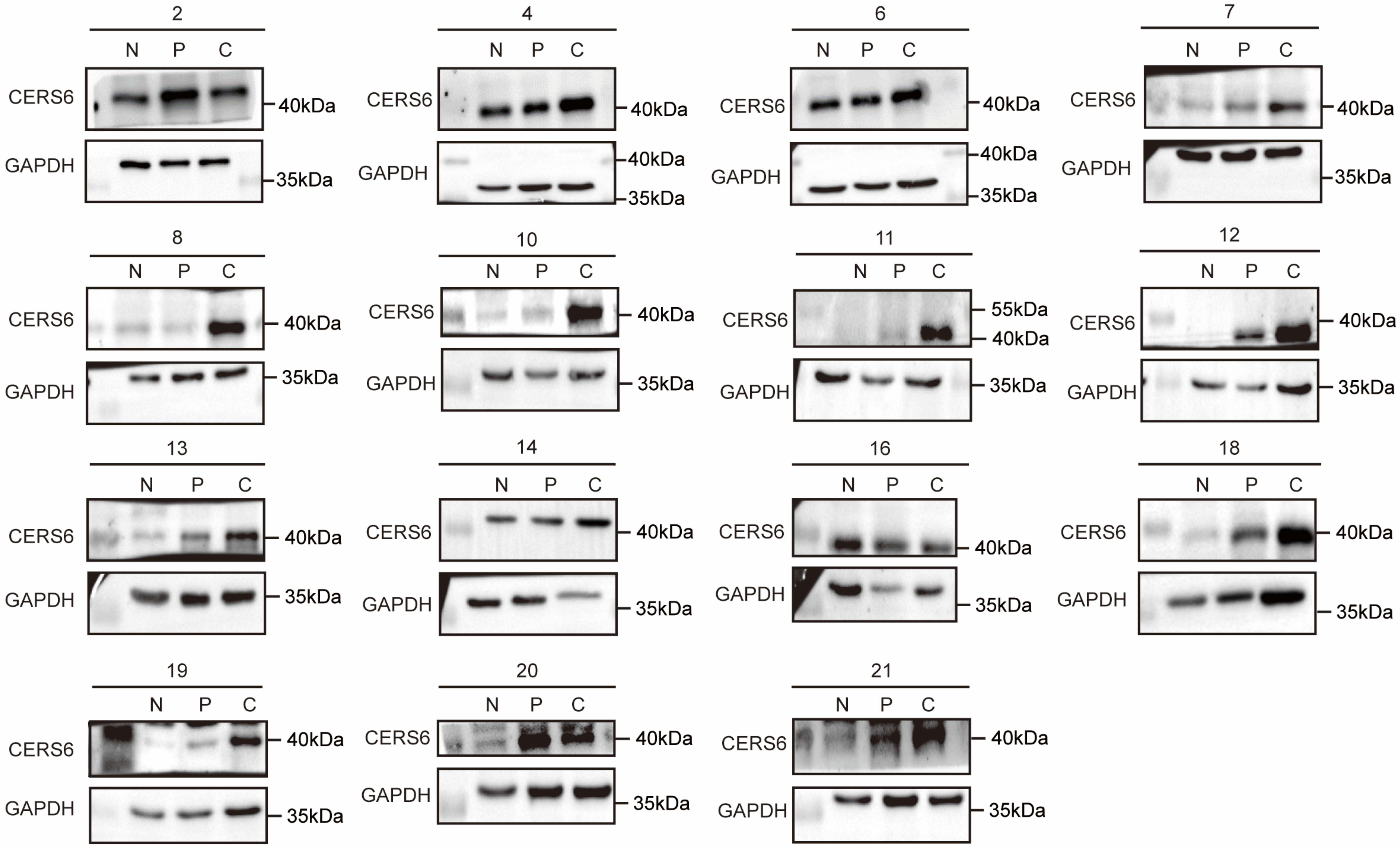

# Figure2 Western blot

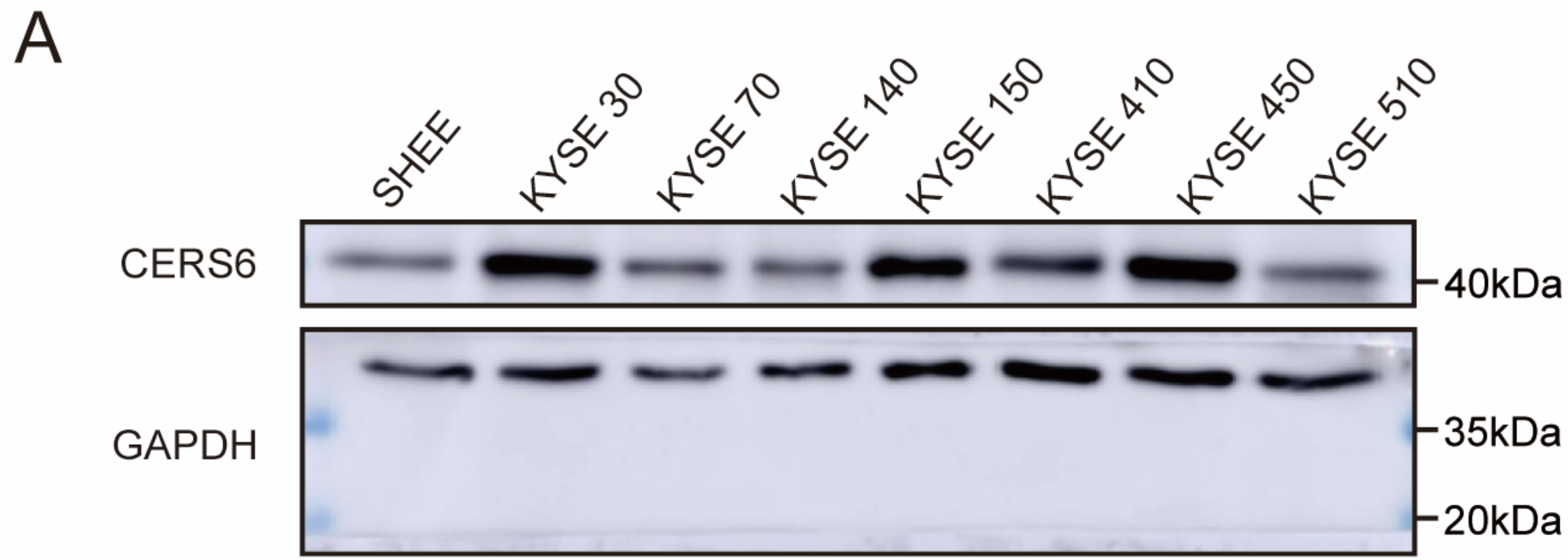


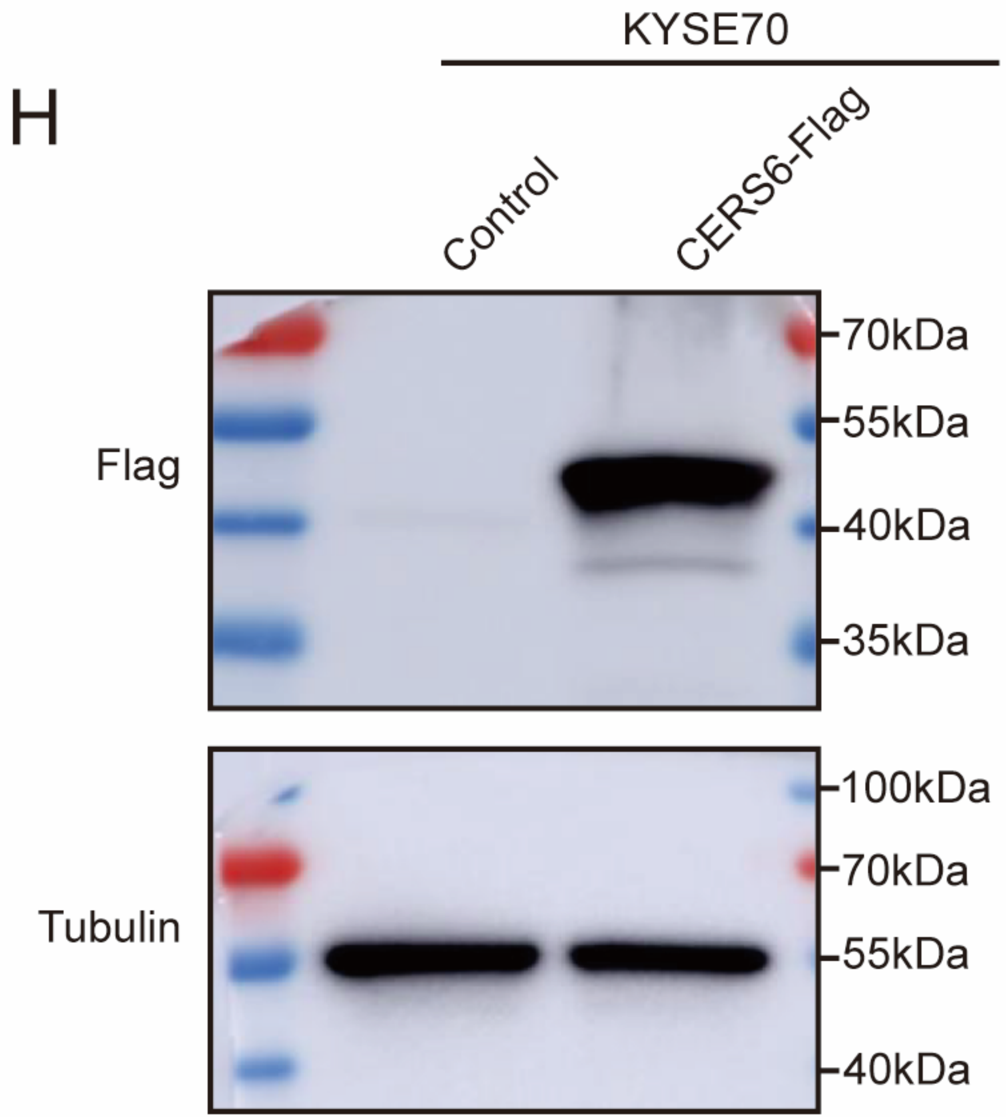

Figure3  Western blot

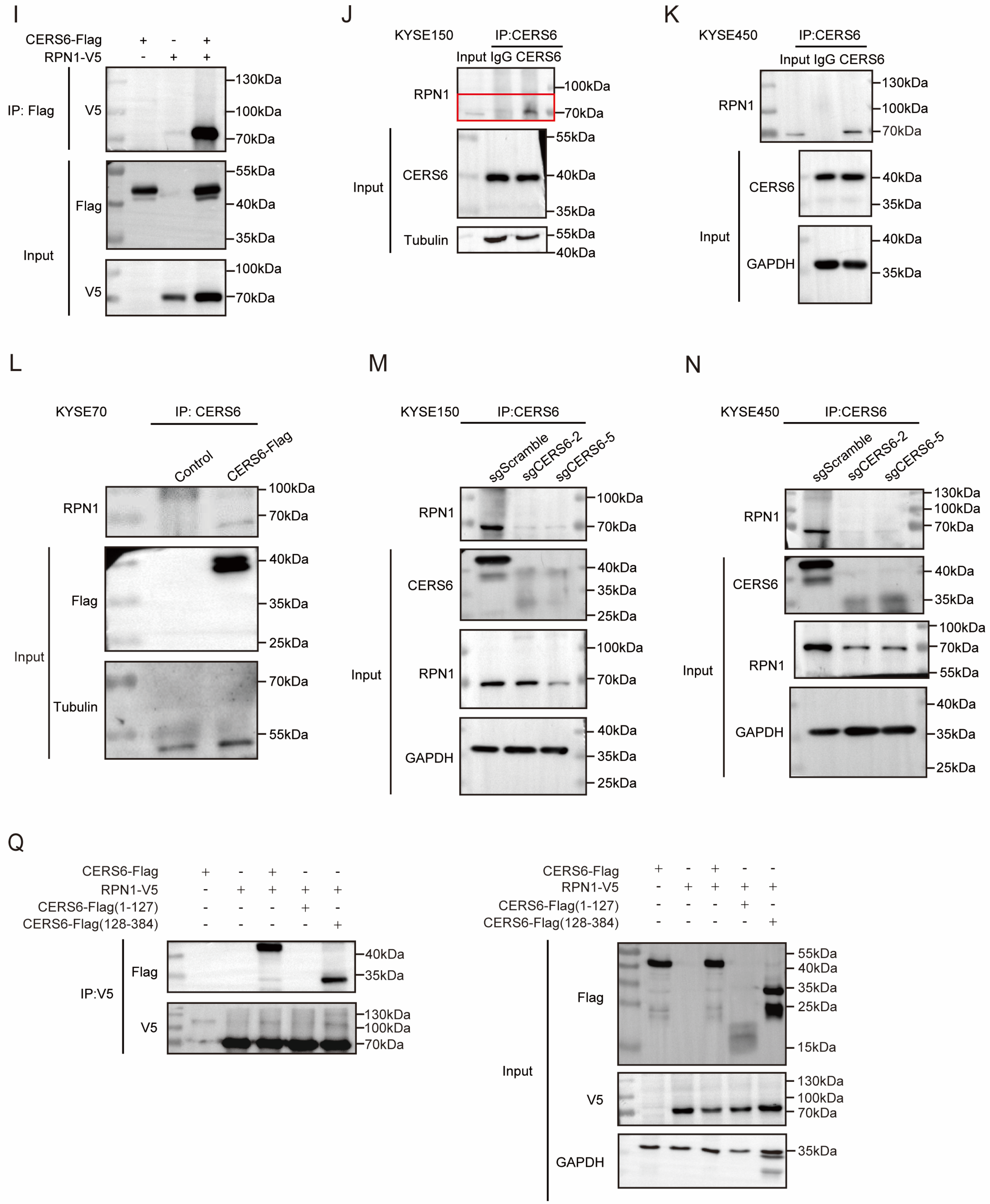

# Figure 4 Western blot

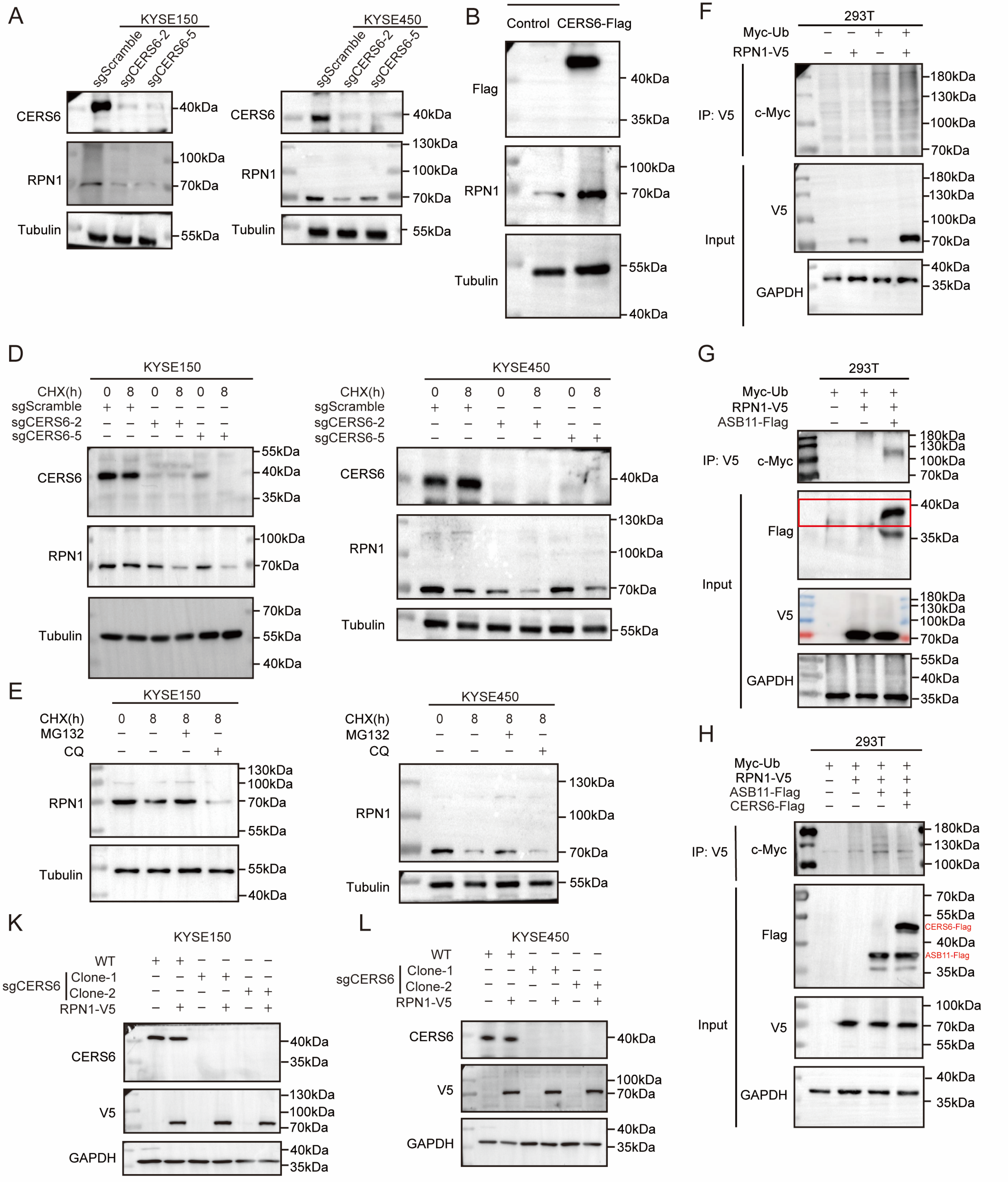

Figure 5 Western blot

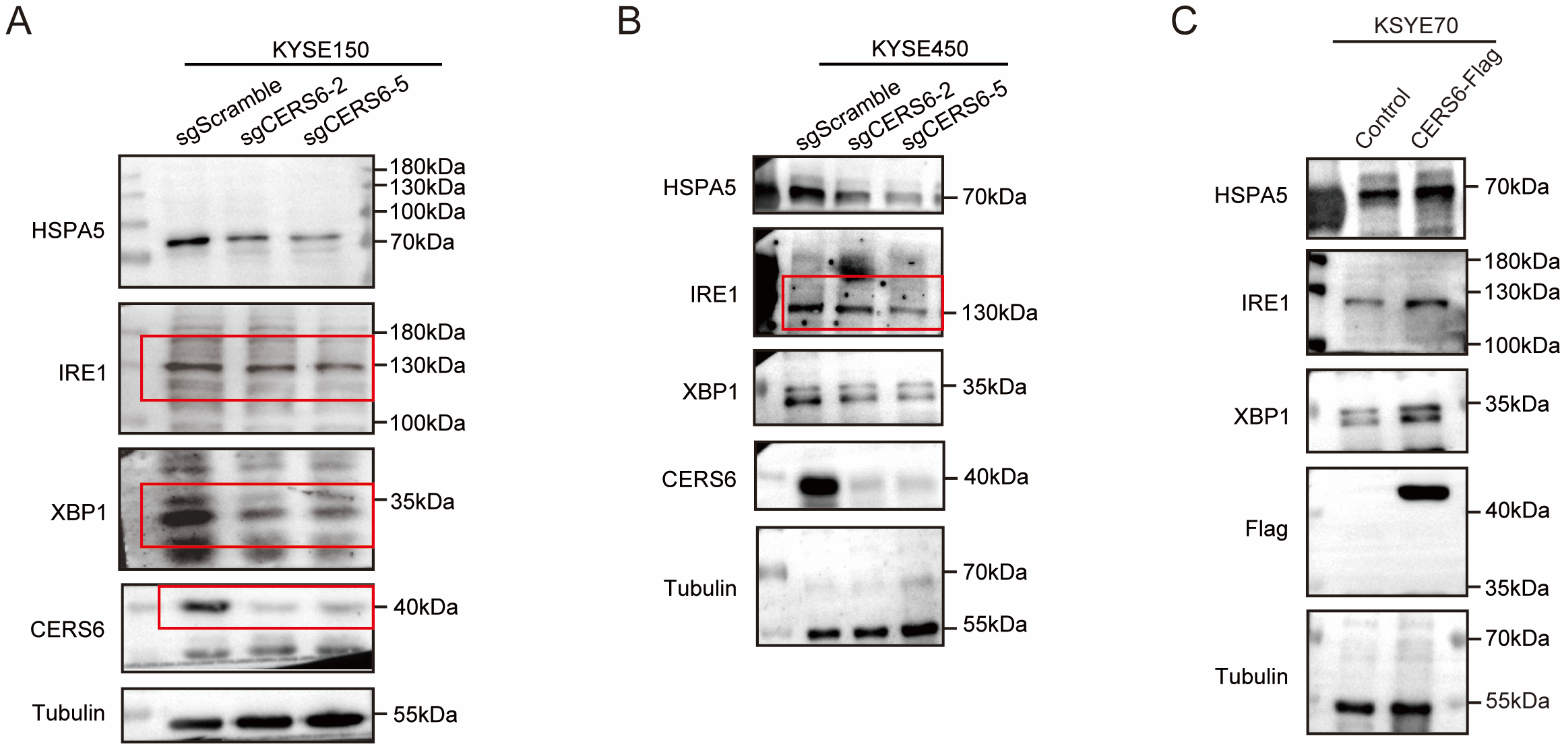

# Figure6 Western blot

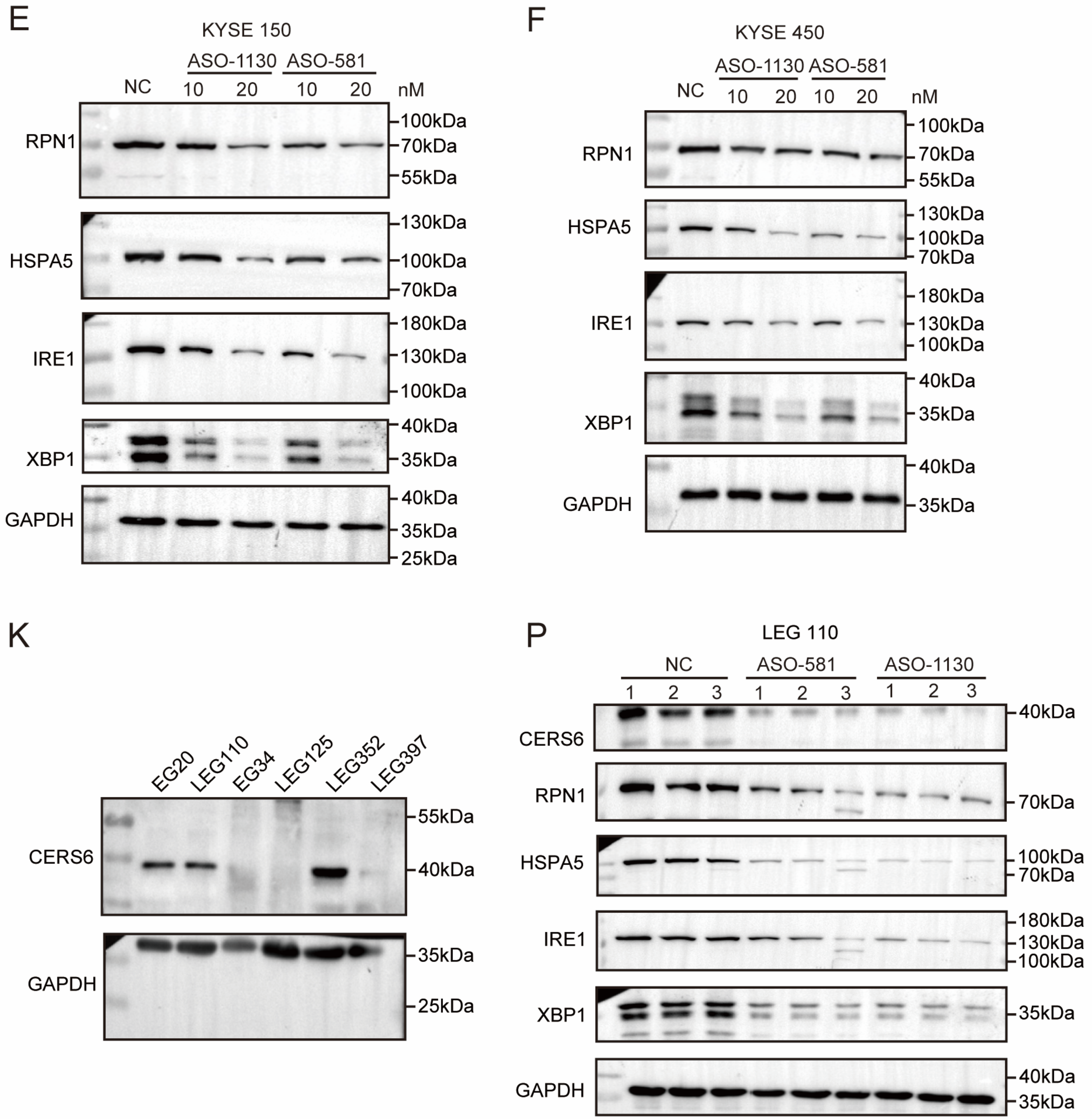

# Figure S1 Western blot

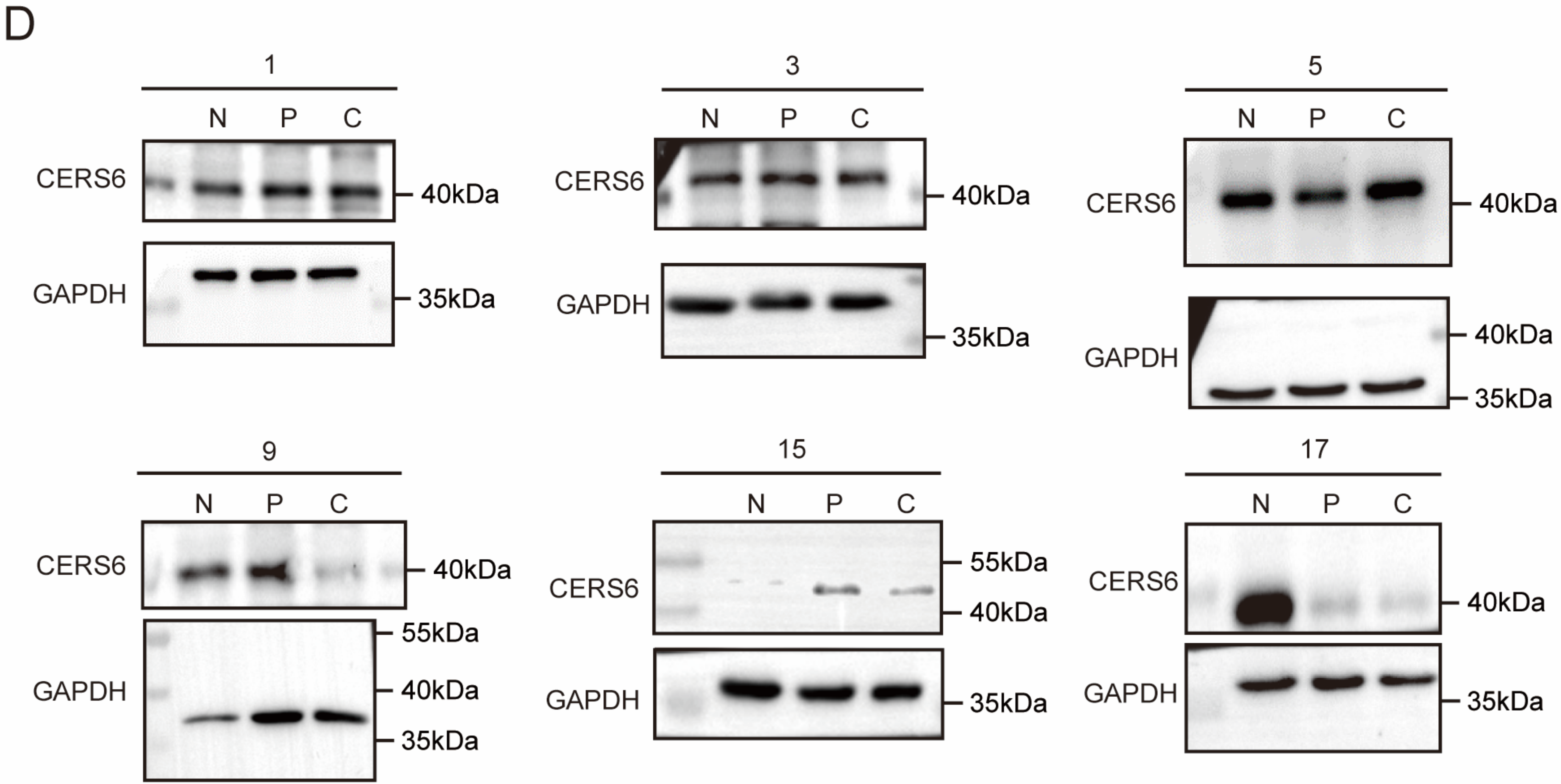

# Figure S2 Western blot

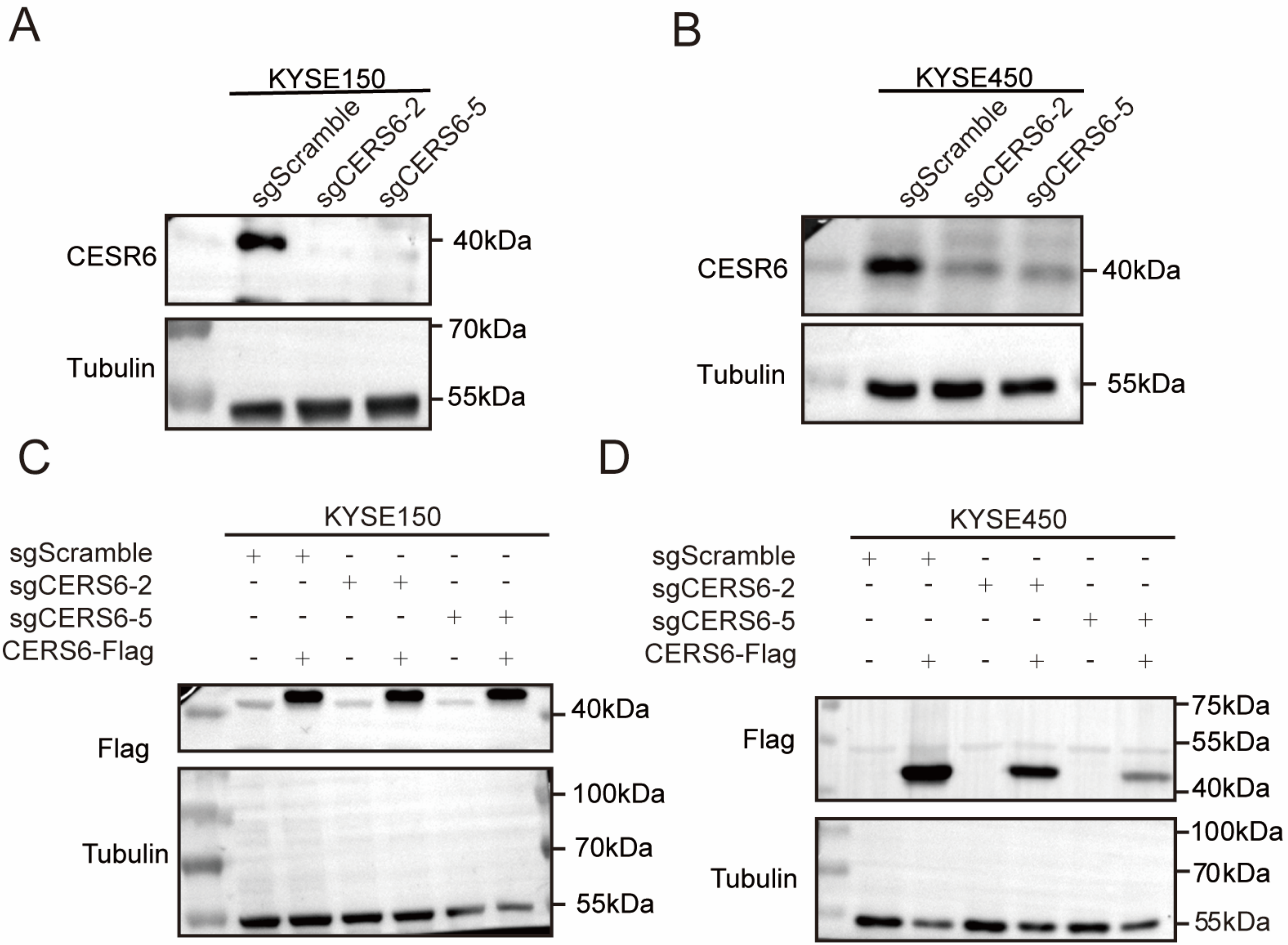

# Figure S4 Western Blot

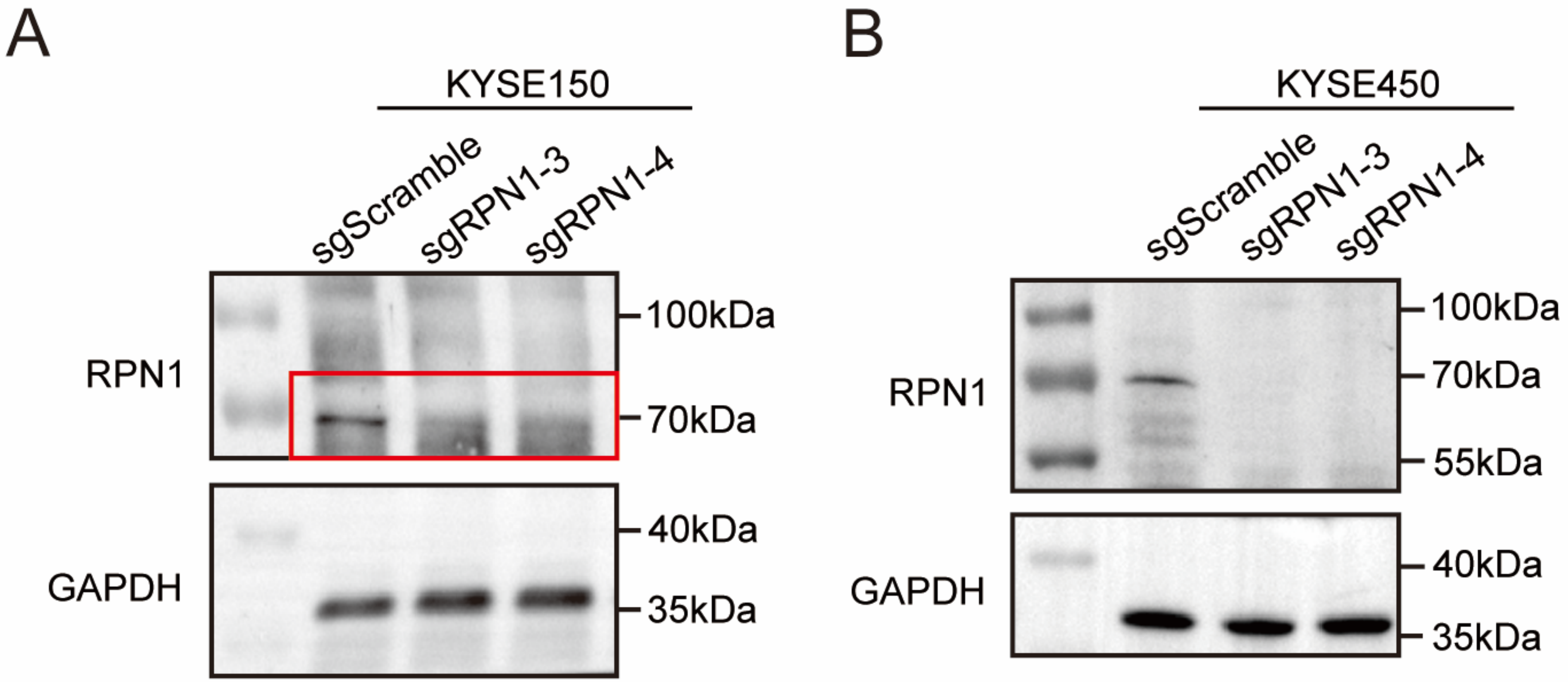

# Figure S6 Western blot

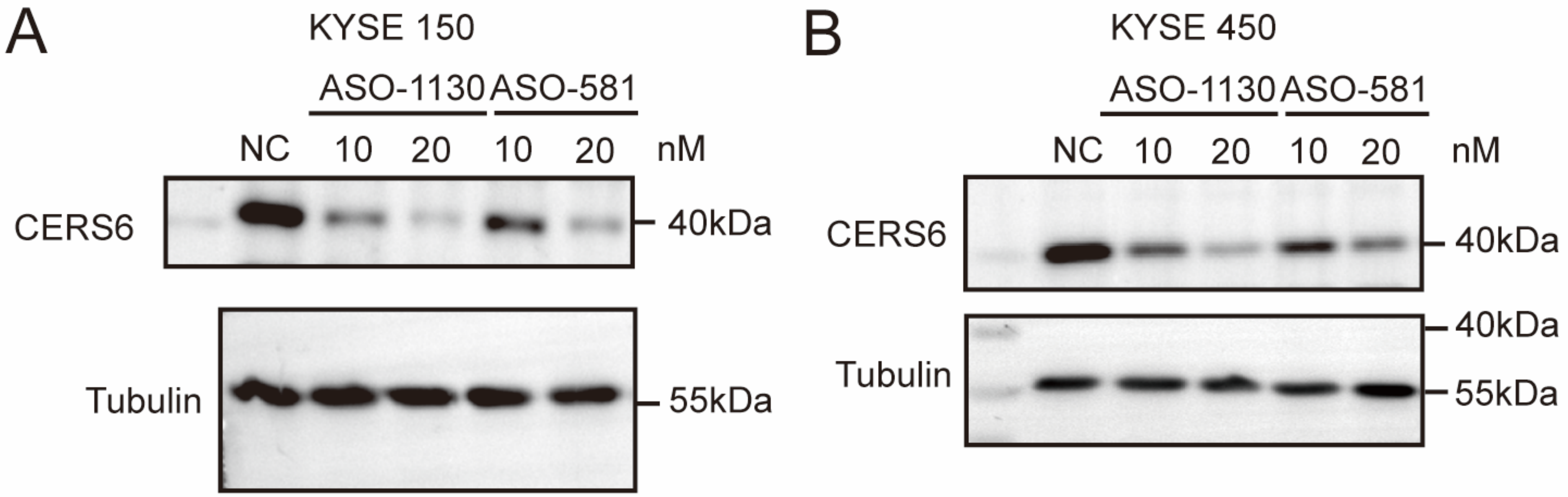

Supplement: Supplementary file 3 — Original Western blots [file 41420_2025_2727_MOESM3_ESM.pdf]
